# ISUP Grade Group Migration Between Prostate Biopsy and Radical Prostatectomy: A Systematic Review of Emerging Predictors

**DOI:** 10.3390/biomedicines14081736

**Published:** 2026-07-31

**Authors:** Victor Pasecinic, Dorin Novacescu, Flavia Zara, Cristina-Stefania Dumitru, Antonia Armega-Anghelescu, Silviu Latcu, Alin Cumpanas, Radu Caprariu, Pavel Banov, Ademir Horia Stana, Radu Gheorghe Dan, Raluca Dumache

**Affiliations:** 1 Doctoral School, Victor Babes University of Medicine and Pharmacy Timisoara, E. Murgu Square, No. 2, 300041 Timisoara, Romania; victor.pasecinic@umft.ro (V.P.); silviu.latcu@umft.ro (S.L.); 2 Department II of Microscopic Morphology, Victor Babes University of Medicine and Pharmacy Timisoara, E. Murgu Square, No. 2, 300041 Timisoara, Romania; flavia.zara@umft.ro (F.Z.); cristina-stefania.dumitru@umft.ro (C.-S.D.); 3 Angiogenesis Research Center, Victor Babes University of Medicine and Pharmacy Timisoara, E. Murgu Square, No. 2, 300041 Timisoara, Romania; 4 Department XV, Discipline of Urology, Victor Babes University of Medicine and Pharmacy Timisoara, E. Murgu Square, No. 2, 300041 Timisoara, Romania; cumpanas.alin@umft.ro; 5 Department XV, Discipline of Radiology and Medical Imaging, University of Medicine and Pharmacy “Victor Babes” Timisoara, E. Murgu Square, Nr. 2, 300041 Timisoara, Romania; 6 Department of Urology and Surgical Nephrology, Nicolae Testemitanu State Medical and Pharmaceutical University, Stefan cel Mare si Sfant Boulevard, No. 165, MD-2004 Chisinau, Moldova; pavel.banov@usmf.md; 7 Department of Medicine, Discipline of Radiology, Vasile Goldiş Western University, Liviu Rebreanu Boulevard, No. 86, 310414 Arad, Romania; stana.ademir@uvvg.ro; 8 Department of Surgery 1, Victor Babes University of Medicine and Pharmacy Timisoara, E. Murgu Square, No. 2, 300041 Timisoara, Romania; radu.dan@umft.ro; 9 Department VIII, Discipline of Forensic Medicine, Bioethics, Deontology and Medical Law, Victor Babes University of Medicine and Pharmacy Timisoara, E. Murgu Square, No. 2, 300041 Timisoara, Romania; raluca.dumache@umft.ro

**Keywords:** prostate cancer, ISUP grade group discordance, ISUP upgrading or downgrading, Gleason score, radical prostatectomy, prostate biopsy, multiparametric MRI, PSMA-PET, genomic classifier, artificial intelligence

## Abstract

**Background/Objectives**: International Society of Urological Pathology (ISUP) Grade Group discordance between prostate biopsy and radical prostatectomy (RP) affects 25–50% of men with localized prostate cancer and has direct implications for active surveillance, nerve-sparing, and adjuvant-treatment decisions. We synthesized contemporary evidence on the frequency of biopsy-to-RP grade migration and on emerging imaging, molecular/genomic, and artificial-intelligence (AI)-based predictors of migration. **Methods**: MEDLINE (PubMed) was searched from 1 January 2010 to 5 May 2026, supplemented by citation searching of recent systematic reviews. Eligible studies reported paired biopsy–RP pathology under the modified 2005 Gleason or the 2014/2019 ISUP Grade Group system, with ≥50 paired cases. Risk of bias was assessed with QUIPS, PROBAST, and QUADAS-2; certainty of evidence was rated with a GRADE framework adapted for prognostic research. Synthesis followed the SWiM reporting guidance. **Results**: Thirty-seven primary studies and eight prior systematic reviews were included. Concordance ranged from 44% to ~70%, upgrading from 14% to 67%, and downgrading from 5% to 26%. Biopsy GG1 disease showed the highest absolute upgrading risk (55–67%). Four predictors reached moderate GRADE certainty: PI-RADS category, biopsy approach (combined vs. systematic), PSMA-PET maximum standardized uptake value, and PSA density (PSAD). Cribriform/intraductal carcinoma at biopsy, the Decipher genomic classifier, the Prostate Health Index, and machine-learning and radiomics models reached very low certainty; clinical nomograms reached low certainty; germline alterations as direct predictors reached very low certainty. PROBAST flagged the analysis domain as high risk in five of eight prediction-model studies, and only one of eight models was externally validated. **Conclusions**: Despite a decade of refinements in grading, imaging, biopsy technique, and molecular profiling, biopsy-to-RP grade discordance remains substantial. Contemporary evidence supports incorporating PI-RADS, biopsy approach, and PSAD into shared decision-making; emerging molecular and AI-based predictors require prospective external validation in adequately powered, geographically representative cohorts before routine clinical adoption.

## 1. Introduction

Prostate cancer is the second most commonly diagnosed malignancy in men worldwide, with approximately 1.47 million new cases and nearly 398,000 deaths estimated globally in 2022, and incidence rates continue to rise in most regions [1]. The clinical behavior of localized prostate cancer is exceptionally heterogeneous, ranging from indolent disease that may safely be managed by active surveillance to aggressive tumors that progress rapidly to metastatic, lethal disease despite definitive local therapy [2,3]. Among the prognostic factors that drive this heterogeneity, histological grade—ascertained on a needle biopsy at the time of diagnosis—has remained, for more than half a century, the single most powerful predictor of cancer-specific outcome and the cornerstone of treatment-selection algorithms [4,5].

The grading of prostate cancer has evolved through three major paradigms. The original Gleason system, introduced in the late 1960s and 1970s, scored cancer aggressiveness from 2 to 10 based on the architectural pattern of the two most prevalent glandular patterns [6]. The 2005 ISUP consensus conference refined pattern definitions and required reporting of the highest, rather than the second most common, pattern [7], and the 2014 ISUP Consensus Conference introduced the five-tier Grade Group (GG) system—GG1 (Gleason ≤ 3 + 3 = 6) through GG5 (9–10)—now adopted by the World Health Organization, the College of American Pathologists, and the AJCC staging manual [8,9,10]. The 2019 ISUP Consensus Conference further mandated reporting of Gleason pattern 4 percentage and of cribriform/intraductal carcinoma (IDC) morphology, both now recognized as independently prognostic [11,12].

Despite these refinements, the clinically critical limitation of needle-biopsy grading has not been resolved: the histological grade reported on the biopsy frequently differs from that ultimately observed on the RP specimen. In a landmark international meta-analysis published in 2008, Cohen and colleagues reported that biopsy and prostatectomy Gleason scores (GSs) were concordant in only 63% of paired cases, with upgrading observed in 30% and downgrading in 7%; in the institutional Lahey Clinic Medical Center component (*n* = 2890) of the same study, concordance was 58%, upgrading 36%, and downgrading 5% [13]. A subsequent Johns Hopkins series of 7643 paired biopsy–RP specimens, using the modified 2005 Gleason scheme, demonstrated that 36.3% of biopsy GS 5–6 tumors were upgraded to ≥GS 7 at surgery and identified increasing age, higher PSA, lower prostate weight, and greater maximum percentage of cancer per core as independent predictors of upgrading [14]. Contemporary registries continue to confirm the persistence of grade migration in the multiparametric magnetic resonance imaging (mpMRI) era: the British Association of Urological Surgeons RP Registry (*n* = 17,598) reported a concordance of 58.9%, upgrading of 25.5%, and downgrading of 15.6% [15], while a recent meta-analysis of 48 studies and 63,119 patients with biopsy GG2 disease reported a median incidence of upgrading to ≥4 + 3 (≥GG3) of 23.4% and of upgrading to ≥GS 8 (≥GG4) of 3.6% [16]. Across contemporary series, biopsy-to-RP grade discordance affects 25–50% of patients [15,17,18].

The clinical consequences of this discordance are substantial. Misclassification of biopsy GG1 disease as eligible for active surveillance may lead to undertreatment of cancers that harbor higher-grade components on whole-mount pathology, with reclassification at confirmatory or surveillance biopsy remaining the principal trigger for definitive intervention in contemporary protocols [19,20]. Conversely, downgrading at RP—which has become more frequent with widespread use of MRI-targeted biopsy, occurring in approximately 15% of contemporary cohorts [21,22]—raises the possibility that some patients are subjected to definitive surgery for disease that might safely have been observed. Beyond active surveillance, biopsy-to-RP grade migration directly influences nerve-sparing decisions, the extent of pelvic lymph-node dissection, eligibility for focal therapy, and the indication for adjuvant radiotherapy, hormonal therapy, or genomic testing [3,5,23]. A substantial proportion of men deemed eligible for focal therapy on biopsy criteria are reclassified as ineligible after final histopathology, although exact estimates vary by cohort and eligibility definition [24].

Three converging lines of evidence have transformed the diagnostic landscape over the past decade and offer the prospect of narrowing the biopsy-to-RP discordance gap. First, mpMRI with Prostate Imaging–Reporting and Data System (PI-RADS) scoring and MRI-targeted, combined, or in-bore biopsy sampling has been shown in multiple randomized trials and meta-analyses (PROMIS, PRECISION, MRI-FIRST, Trio Study, STHLM3-MRI, and GÖTEBORG-2) to improve the detection of clinically significant prostate cancer and to reduce, although not eliminate, biopsy-to-RP upgrading; prostate-specific membrane antigen positron emission tomography (PSMA-PET), with maximum standardized uptake value (SUV_max_) and the PRIMARY score, has shown a robust monotonic relationship with RP grade [22,25,26,27,28]. Second, tissue-based genomic classifiers (Decipher, Prolaris, Oncotype DX Genomic Prostate Score), urinary biomarkers (ExoDx EPI, MyProstateScore, SelectMDx), serum biomarkers (Prostate Health Index [PHI], 4Kscore), and germline assessment of DNA-damage-repair genes (*BRCA1/2*, *ATM*, *HOXB13*, *CHEK2*, *MSH2*) have begun to refine risk stratification beyond standard clinicopathological variables [29,30,31,32]. Third, deep-learning algorithms applied to digital histopathology (Paige Prostate, Radboud/Karolinska systems, the PANDA challenge consortium) and to mpMRI radiomics have achieved pathologist-level performance in Gleason grading and have begun to demonstrate measurable reductions in upgrading and downgrading between biopsy and RP [33,34,35,36]. The optimal way to integrate these emerging predictors with established clinical variables, however, remains unsettled, and the comparative evidence for their effect on biopsy-to-RP grade migration has not, to our knowledge, been the subject of a comprehensive Preferred Reporting Items for Systematic Reviews and Meta-Analyses (PRISMA) 2020-compliant systematic review.

Several existing systematic reviews have addressed components of this question—for example, the impact of mpMRI-targeted biopsy on grade concordance [22,27], the predictive value of PSMA-PET SUV_max_ [28], or specific clinical predictors of GS upgrading [37]. To date, however, no review has synthesized evidence across the full spectrum of emerging predictors—imaging, molecular/genomic, and AI-based—under a single, methodologically rigorous PRISMA 2020 framework that incorporates dedicated risk-of-bias assessment for prognostic-factor (Quality In Prognosis Studies [QUIPS]) and prediction-model (Prediction model Risk Of Bias Assessment Tool [PROBAST]) studies and applies a Grading of Recommendations Assessment, Development and Evaluation (GRADE) adaptation for prognostic certainty. Such a synthesis is increasingly needed, as guidelines (the EAU-EANM-ESTRO-ESUR-ISUP-SIOG 2024 update, the NCCN Prostate Cancer Guidelines v.3.2024) integrate new biomarkers into routine practice and as patients and clinicians confront treatment decisions of growing complexity [3,5].

Accordingly, the aims of this systematic review are to (i) quantify the contemporary prevalence of upgrading and downgrading between prostate biopsy and RP across the modified 2005 Gleason and 2014/2019 ISUP GG eras; (ii) identify and synthesize the evidence base for emerging imaging predictors (PI-RADS category, mpMRI radiomics, PSMA-PET parameters), molecular predictors (tissue genomic classifiers, urinary and serum biomarkers, germline alterations, cribriform/intraductal morphology), and AI-based predictors (deep-learning algorithms applied to digital pathology and to MRI) of grade migration; (iii) compare the magnitude of effect, methodological quality, and certainty of evidence for these emerging predictors against that of established clinical predictors (PSA, PSA density [PSAD], percent positive cores, perineural invasion, biopsy approach); (iv) appraise the discrimination, calibration, and external validity of multivariable prediction models for biopsy-to-RP grade migration; and (v) identify evidence gaps and prioritize the emerging predictors most likely to inform next-generation, multimodal risk-stratification tools. The corresponding research questions are framed in the Population, Index prognostic factors, Comparator, Outcomes, Timing, and Setting (PICOTS) structure (detailed below in Section 2.2).

## 2. Materials and Methods

This systematic review was conducted and is reported in accordance with the PRISMA 2020 statement [38]. Because the review addresses prognostic factors and prediction models rather than a single intervention, the PRISMA 2020 framework was supplemented by reporting guidance specific to prognostic-factor reviews and, where relevant, by the Checklist for Critical Appraisal and Data Extraction for Systematic Reviews of Prediction Modelling Studies (CHARMS) [39]. A completed PRISMA 2020 checklist accompanies this manuscript as a Supplementary File.

### 2.1. Protocol and Registration

The review protocol was developed a priori following the PRISMA-P recommendations and was registered prospectively on the International Prospective Register of Systematic Reviews (PROSPERO; registration number CRD420261378765). No ethical approval or patient consent was required because the review synthesizes data from previously published studies.

### 2.2. Eligibility Criteria and Review Question

The review question was framed using a PICOTS structure adapted for a prognostic-factor and prediction-model review. The population comprised adult men (≥18 years) with biopsy-diagnosed, clinically localized or locally advanced non-metastatic prostate adenocarcinoma who subsequently underwent RP without neoadjuvant androgen-deprivation, radiation, or systemic therapy. Index factors included any clinical, biochemical, imaging, pathological, molecular/genomic, or artificial-intelligence (AI)-derived predictor; emerging predictors of special interest were pre-specified as mpMRI with PI-RADS assessment, mpMRI- and PET-based radiomics, PSMA-PET parameters (SUV_max_, PRIMARY score), tissue-based genomic classifiers (Decipher, Prolaris, Oncotype DX GPS), urinary biomarkers (SelectMDx, ExoDx/EPI, MyProstateScore, PCA3), serum biomarkers (PHI, 4Kscore), germline and somatic alterations in DNA-damage-repair and prostate-cancer-susceptibility genes (*BRCA1/2*, *ATM*, *HOXB13*, *CHEK2*, *MSH2*, *PTEN*), cribriform and IDC morphology, and AI/deep-learning algorithms applied to digital histopathology or MRI. Comparators were participants without the predictor (or with lower values) for single-factor studies as well as standard clinicopathological models (Epstein criteria, D’Amico, CAPRA, or existing nomograms) for prediction-model studies.

The primary outcome was ISUP GG migration between biopsy and RP, operationalized as upgrading (≥1 GG increase) or downgrading (≥1 GG decrease). Secondary outcomes included clinically significant upgrading (defined a priori as any increase to GG ≥2 from biopsy GG1 or to GG ≥ 3 from biopsy GG2), concordance rates, prognostic-factor effect estimates (ORs, HRs, regression coefficients with 95% CIs), and prediction-model performance metrics (AUC/C-index, calibration intercept and slope, decision-curve net benefit). Studies using the modified 2005 ISUP Gleason system or the 2014/2019 ISUP GG system were eligible; studies relying exclusively on pre-2005 Gleason grading were excluded. The setting comprised academic, tertiary, and community institutions worldwide.

Because the included studies used heterogeneous definitions of grade migration, we applied a pre-specified standardized framework to interpret and tabulate every cohort on a common scale. (i) The reference outcome throughout this review is any-increase upgrading, defined as an increase of at least one ISUP GG from the index biopsy to the RP specimen (and, symmetrically, any-decrease downgrading); this is the most consistently reportable definition across eras and is the value entered in our report on the characteristics and primary outcome rates of included studies (see Section 3.2), unless a footnote states otherwise. (ii) Clinically significant upgrading was analyzed separately and defined a priori as crossing a management-relevant threshold—any increase to GG ≥ 2 from biopsy GG1 (the active-surveillance-relevant transition) or to GG ≥ 3 from biopsy GG2 (the nerve-sparing/treatment-intensity-relevant transition); where a primary study reported only this clinically significant definition, it is indicated explicitly in the report footnotes (see Section 3.2; for example, the GG1 → GG ≥ 2 transition in Su et al. [40] and Liss et al. [41] and the GS5–6 → ≥ GS7 transition in Epstein et al. [14]). (iii) To harmonize grading-system differences, modified 2005 Gleason-score data were mapped onto the five-tier GG system using the standard correspondence (GS ≤ 6 = GG1; 3 + 4 = GG2; 4 + 3 = GG3; 8 = GG4; 9–10 = GG5); where a study reported both systems on the same cohort (for example De Nunzio et al. [42]), both figures are retained, and the GG-system value is used in synthesis. Studies reporting only a dichotomous “significant-versus-not” outcome were not force-fitted into the five-tier scale but were synthesized within the clinically significant upgrading stratum. We did not attempt to re-derive grade from raw pattern data, and residual definitional heterogeneity is one reason the reported upgrading range (14–67%) is wide; this is examined explicitly in the subgroup synthesis (Section Subgroup and Sensitivity Synthesis) and is the principal stated reason for which quantitative pooling was not undertaken (Section 2.6).

Studies were eligible if they were original peer-reviewed observational (prospective or retrospective cohort, case-control) studies or clinical trials published between 1 January 2010 and the date of final search; enrolled adult men with histologically confirmed prostate adenocarcinoma diagnosed by transrectal or transperineal biopsy (systematic, MRI-targeted, combined, or in-bore) followed by RP; reported at least one measure of GG concordance, upgrading, or downgrading, the association of ≥1 predictor with GG migration, or the development/validation of a prediction model; included ≥50 paired biopsy–RP cases; and were available in full text in English. Studies were excluded if they reported only biopsy or only RP pathology without paired comparison; enrolled patients receiving neoadjuvant systemic therapy, primary radiotherapy, focal therapy, or active surveillance without subsequent RP; relied exclusively on pre-2005 Gleason data; included fewer than 50 paired cases; were case reports, editorials, narrative reviews, letters, or conference abstracts without an accessible full-text manuscript; substantially overlapped with a larger included cohort from the same institution (in which case, the most recent or largest report was retained); or could not be retrieved in full text after two contact attempts to the corresponding author.

### 2.3. Information Sources and Search Strategy

An electronic search was performed in MEDLINE via PubMed; no other bibliographic databases (Embase, the Cochrane Central Register of Controlled Trials, Scopus, Web of Science) were searched, and no preprint servers, trial registries, or conference proceedings were systematically interrogated. Citation searching of recent relevant systematic reviews and meta-analyses was used to identify a small number of additional records that were not retrieved by the PubMed query. The search covered the period from 1 January 2010 to 5 May 2026 (final search update). Restricting information sources to a single bibliographic database is a known threat to search sensitivity and is acknowledged as a limitation of this review (see Section 4.4).

The PubMed search strategy combined Medical Subject Headings (MeSH) terms with free-text terms drawn from three conceptual blocks, linked by AND with synonyms within each block linked by OR: (1) the population (prostate adenocarcinoma and RP); (2) the grading system and migration event (Gleason, GG, ISUP, upgrading, downgrading, discordance, concordance, reclassification, upstaging); and (3) the diagnostic or prognostic intervention (biopsy, mpMRI, PI-RADS, PSMA-PET, genomic classifiers, AI/deep learning, radiomics, biomarkers of interest). Searches were restricted to English-language publications. PubMed-native truncation and field tags were used; no methodological filters that might exclude observational designs were applied. The complete electronic search strategy—including the exact MeSH headings, free-text terms, Boolean operators, truncation symbols, and field tags used within each conceptual block, together with the verbatim PubMed query string and block-level hit counts—is provided in Supplementary File S1, allowing the search to be independently re-run and audited.

Restricting the primary search to MEDLINE/PubMed is the principal methodological limitation of this review. Single-database searching reduces sensitivity, and the resulting omissions are unlikely to be random across predictor domains: studies of MRI radiomics and artificial intelligence are disproportionately reported in engineering, imaging-informatics, and conference-indexed venues that are captured more completely by Embase, IEEE Xplore, Scopus, and Web of Science than by MEDLINE, making the emerging-predictor literature the domain most susceptible to under-sampling. Citation searching of recent systematic reviews and meta-analyses partially mitigates this gap but is not a substitute for systematic multi-database searching. The present review is therefore best regarded as a contemporary MEDLINE-anchored synthesis, and the certainty ratings and the strength of the conclusions drawn for AI- and radiomics-based predictors are interpreted with corresponding caution (Section 4.4 and Section 4.5).

### 2.4. Study Selection and Data Extraction

Retrieved records were exported to EndNote 21 (Clarivate, Philadelphia, PA, USA) for deduplication and uploaded to Rayyan (Rayyan Systems Inc., Cambridge, MA, USA; https://www.rayyan.ai, accessed on 25 April 2026) for screening. Title-and-abstract screening and full-text eligibility assessment were performed independently and in duplicate by two reviewers, who screened records in parallel and were blinded to each other’s decisions during screening. Disagreements at each stage were resolved by discussion, with provision for adjudication by a third reviewer if consensus could not be reached. Reasons for full-text exclusion were recorded and are reported in the PRISMA 2020 flow diagram (see Section 3.1).

Data were extracted independently and in duplicate by two reviewers using a pre-piloted, standardized data-extraction form developed in Microsoft Excel (Microsoft 365; Microsoft Corporation, Redmond, WA, USA) and tested on a sample of included studies before full extraction. When multiple reports from the same cohort were identified, data were extracted from the most complete and most recent publication, and overlapping reports were cross-referenced but not double-counted. For each study, we extracted study characteristics (first author, year, country, design, enrolment period, sample size, funding); population characteristics (age, race/ethnicity, PSA, PSAD, prostate volume, clinical T stage, risk-group distribution); biopsy characteristics (route, technique, number of cores, percent positive cores, greatest percentage of cancer per core, central versus local pathology review, pathologist expertise); imaging characteristics (mpMRI field strength and PI-RADS version, radiologist experience, PSMA-PET tracer and protocol, radiomics pipeline); molecular/genomic characteristics (specific assay, tissue source, scoring thresholds); RP characteristics (surgical approach, specimen handling, pathologist expertise); primary and secondary outcome data (concordance, upgrading, and downgrading proportions stratified by biopsy GG; effect estimates with 95% CIs; prediction-model performance metrics); and methodological characteristics (handling of missing data, multivariable model covariates, validation strategy, adherence to Transparent Reporting of a multivariable prediction model for Individual Prognosis or Diagnosis [TRIPOD] or TRIPOD+AI). Only data reported in the published full text or its Supplementary Materials were extracted; corresponding authors were not contacted to obtain additional or unpublished data. Discrepancies between the two reviewers’ extracted values were resolved by discussion against the source publication and, where necessary, by adjudication by a third reviewer.

### 2.5. Risk of Bias Assessment

Risk of bias in individual studies was assessed independently and in duplicate by two reviewers using instruments appropriate to the study question. For studies investigating a single prognostic factor, the QUIPS tool was applied, evaluating six domains—study participation, attrition, prognostic-factor measurement, outcome measurement, confounding, and statistical analysis and reporting—each rated as low, moderate, or high risk of bias [43]. For studies developing, updating, or validating a multivariable prediction model, PROBAST was used, evaluating four domains (Participants, Predictors, Outcome, Analysis) via 20 signaling questions, with an overall judgement of low, high, or unclear risk of bias and applicability [44]. For studies evaluating the diagnostic accuracy of a test (for example, mpMRI-targeted biopsy or PSMA-PET) against RP whole-mount pathology, the Quality Assessment of Diagnostic Accuracy Studies (QUADAS-2) tool was applied [45]. Inter-rater agreement statistics were not formally computed; disagreements in domain-level and overall judgements were resolved by discussion against the source publication, with provision for adjudication by a third reviewer if consensus could not be reached.

Tool assignment was based on the question each study was being used to answer in our synthesis rather than on the study’s own self-description and was decided a priori by the same two reviewers. A study contributing an adjusted association between a single factor (for example PSAD, PI-RADS category, or cribriform/IDC) and grade migration was appraised with QUIPS; a study developing or validating a multivariable model that outputs an individualized prediction was appraised with PROBAST; and a study reporting the diagnostic accuracy of an index test against RP whole-mount pathology as the reference standard was appraised with QUADAS-2. Several imaging studies legitimately span more than one paradigm—for example, an mpMRI study can yield both a prognostic-factor association (PI-RADS as a predictor) and a diagnostic-accuracy estimate (PI-RADS versus whole-mount). For such studies, the instrument matching the specific estimate extracted was applied, and where more than one type of estimate was extracted, more than one instrument was applied. The corresponding signaling-question-level judgements underpinning these decisions are recorded in the data-extraction file and summarized graphically. Risk-of-bias judgements are detailed below (in Section 3.11).

### 2.6. Data Synthesis and Certainty of Evidence

The principal effect measures were prevalence proportions (for upgrading, downgrading, and concordance), odds ratios (ORs) with 95% CIs (for binary predictor associations), and AUC/C-index with 95% CIs (for prediction-model discrimination). Hazard ratios reported for downstream outcomes (for example, biochemical recurrence) were extracted and summarized narratively but not pooled with ORs. For continuous predictors, adjusted ORs per pre-specified unit of increase were preferred; when only unadjusted estimates were reported, both were extracted, and the adjusted estimate was prioritized in synthesis.

Given the anticipated heterogeneity across studies in biopsy technique, imaging protocol, pathology review, predictor definitions (e.g., upgrading defined as any increase versus increase to GG ≥ 2 or ≥3), and risk-group case-mix, no quantitative meta-analyses were performed in this review. Effect estimates were extracted as reported by the primary studies (proportions for concordance, upgrading, and downgrading; ORs or HRs with 95% CIs for predictor associations; AUC or C-index with 95% CIs for prediction-model discrimination) and synthesized narratively in accordance with the Synthesis Without Meta-analysis (SWiM) reporting guidance [46], with structured tabular summaries of effect direction, magnitude, precision, and risk of bias. Where contemporary published systematic reviews and meta-analyses had already pooled comparable studies for a given predictor (notably biopsy approach and PI-RADS category), their pooled estimates are reported alongside the primary-study findings and are clearly attributed to those reviews rather than re-derived here. Pre-specified narrative subgroups, used to organize synthesis rather than as formal statistical comparisons, included biopsy approach (systematic vs. MRI-targeted vs. combined; transrectal vs. transperineal; in-bore vs. fusion), ISUP grading era (2005 modified Gleason vs. 2014 GG vs. 2019 GG), biopsy GG at diagnosis, central versus local pathology review, and geographic region.

De novo meta-analysis was not undertaken for the three predictors with the most data—PSAD, PI-RADS category, and biopsy approach—for reasons specific to each. For PSAD, primary studies reported the association on incompatible scales (adjusted OR per 0.1 ng/mL/mL increase, OR per 1-unit increase, dichotomies at locally chosen thresholds of 0.10, 0.12, 0.15, and 0.20 ng/mL/mL, and standardized mean differences), and the covariate sets entering the multivariable models differed substantially; pooling these without patient-level data would combine non-equivalent quantities and produce a spuriously precise summary. For PI-RADS, studies mixed PI-RADS versions 2.0 and 2.1, used different reference categories (≤2 vs. ≤3 vs. category-by-category), and applied different reader-experience and field-strength conditions, so a single pooled OR would mask the very heterogeneity that is clinically important. For biopsy approach, the comparison of interest (systematic vs. targeted vs. combined; transrectal vs. transperineal) has already been formally pooled in two contemporary, methodologically robust meta-analyses [22,27] on overlapping study sets; re-pooling the same primary studies here would duplicate that work while risking double-counting. For these predictors, the existing published pooled estimates are therefore reported and attributed explicitly to their source rather than re-derived. To keep the provenance of every quantitative statement transparent, three tiers of evidence are distinguished throughout the Results and Discussion: (a) effect estimates taken directly from an included primary study (named with its first author and sample size); (b) pooled estimates extrapolated from a previously published meta-analysis (named as such and attributed to the source review, e.g., Wang Y et al. [37], Weinstein et al. [22], Goel et al. [27], Chen DC et al. [28], Wu et al. [16]); and (c) qualitative conclusions reached by narrative synthesis across studies. Where the text states a pooled figure, the tier-(b) source is cited, and such estimates are not presented as though they were generated by the present review.

Because no quantitative meta-analyses were performed, the formal statistical assessment of publication or reporting bias (e.g., funnel plots, Egger’s regression test, trim-and-fill) was not applicable; the potential for selective reporting in the underlying primary studies and in the published systematic reviews whose pooled estimates are cited is acknowledged narratively. Certainty of evidence for each predictor–outcome pair was rated independently by two reviewers using the GRADE framework adapted for prognostic-factor research, with certainty categorized as high, moderate, low, or very low across the domains of risk of bias, inconsistency, indirectness, imprecision, publication bias, and phase of investigation; disagreements were resolved by discussion. The GRADE summary of findings is reported below (Section 3.12).

### 2.7. Software

Bibliographic records were managed in EndNote 21 (Clarivate, Philadelphia, PA, USA), screening was performed in Rayyan (Rayyan Systems Inc., Cambridge, MA, USA; https://www.rayyan.ai, accessed on 25 April 2026), and data extraction was performed in Microsoft Excel (Microsoft 365; Microsoft Corporation, Redmond, WA, USA). Figures, including the PRISMA 2020 flow diagram and the visual summaries of biopsy-approach effect estimates, PSMA SUV_max_ distributions, and clinical-predictor associations, were generated in Python (version 3.11) using the matplotlib library (v3.10; The Matplotlib Development Team, NumFOCUS, Austin, TX, USA). No quantitative meta-analysis software (e.g., R meta, metafor, or dmetar) was used, and risk-of-bias visualization software (e.g., R robvis) was likewise not employed. Risk-of-bias judgements were recorded directly in the data-extraction spreadsheet and rendered as detailed below (in Section 3.11). The data-extraction file is available from the corresponding author on reasonable request.

## 3. Results

### 3.1. Study Selection

**Figure 1:**
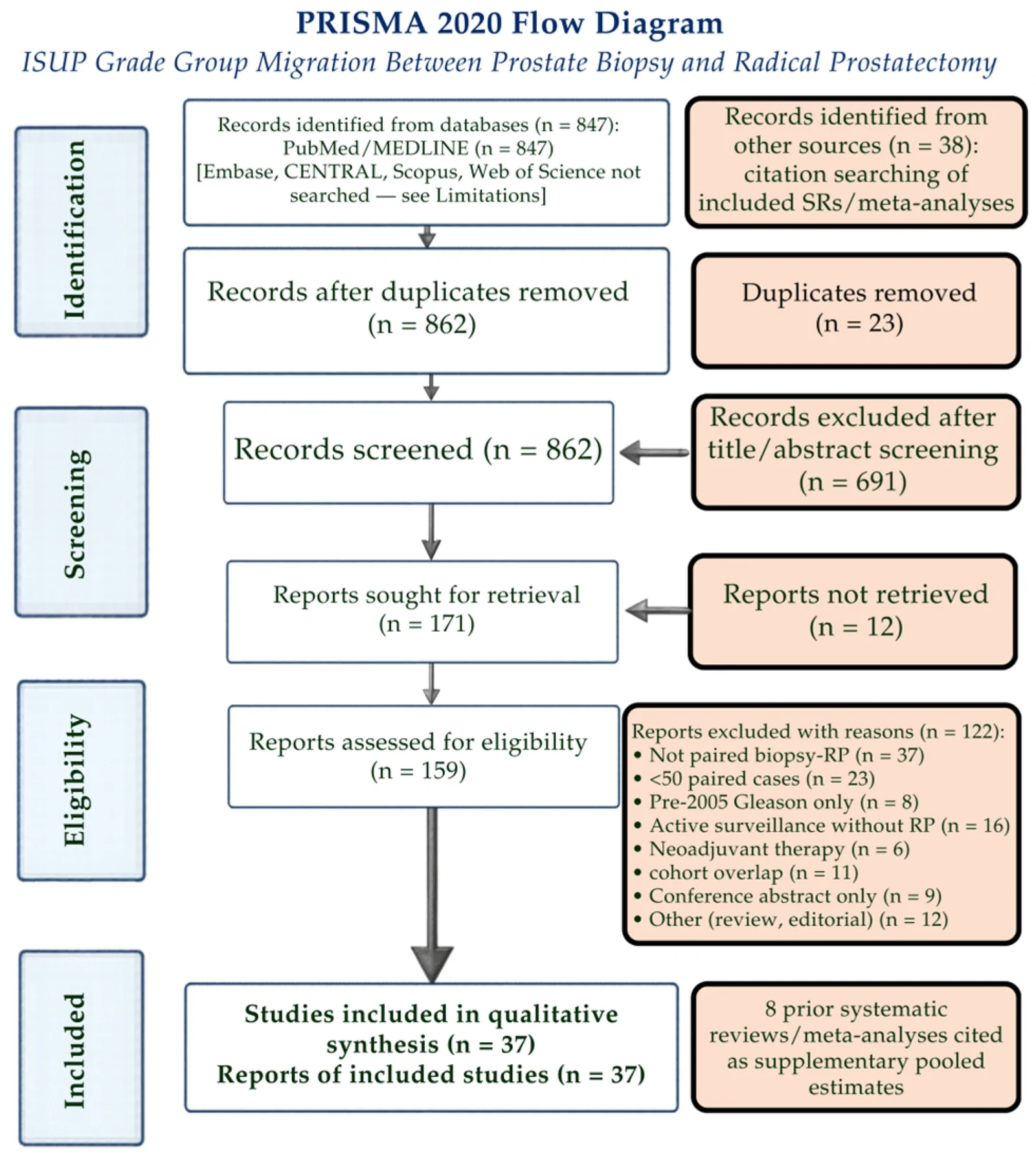
PRISMA 2020 flow diagram for study selection. Records were identified from a single-database PubMed/MEDLINE search (*n* = 847) supplemented by citation searching of recent systematic reviews and meta-analyses (*n* = 38); Embase, CENTRAL (Cochrane Central Register of Controlled Trials), Scopus, and Web of Science were not searched (see Section 4.4 for limitations). After removal of 23 duplicates, 862 records were screened by title and abstract, of which 691 were excluded. Of the 171 reports sought for retrieval, 12 could not be obtained in full text in English; 159 reports were assessed for eligibility against the inclusion criteria, and 122 were excluded with reasons as shown. Thirty-seven primary studies were included in the qualitative synthesis. The side annotation on the right indicates that pooled estimates from eight contemporary systematic reviews and meta-analyses [13,16,22,27,28,37,47,48] are reported alongside the primary-study findings; these reviews are not additional records that flowed through the identification, screening, and eligibility stages of the search. Abbreviations: PRISMA = Preferred Reporting Items for Systematic Reviews and Meta-Analyses; RP = radical prostatectomy; SR = systematic review.

The flow of records through the review is summarized in Figure 1. PubMed/MEDLINE searching identified 847 records, supplemented by 38 records identified through citation searching of relevant systematic reviews and meta-analyses. After removal of 23 duplicates, 862 records were screened by title and abstract; 691 were excluded as off-topic, of ineligible design (narrative review, editorial, animal or in vitro study), or clearly outside the population of interest. Of the 171 reports sought for retrieval, 12 could not be obtained in full text in English. Of the 159 full-text reports assessed against the eligibility criteria, 122 were excluded with reasons (Figure 1), most commonly because biopsy and RP pathology were not paired (*n* = 37), the cohort comprised <50 paired cases (*n* = 23), the report described an active-surveillance cohort without subsequent RP (*n* = 16), or there was substantial cohort overlap with a larger included study from the same institution (*n* = 11). Thirty-seven primary studies met all eligibility criteria and were included in the qualitative synthesis. Eight contemporary systematic reviews and meta-analyses [13,16,22,27,28,37,47,48] are cited as supplementary pooled estimates alongside the primary-study findings; these are shown as a side annotation in Figure 1 rather than as additional records flowing through the search, since they were not subjected to the eligibility criteria applied to primary studies. Prior reviews were treated as supplementary regardless of publication date and were therefore not subject to the post-2010 cut-off applied to primary studies; this is why the Cohen et al. 2008 international meta-analysis [13] is cited despite predating the primary-study window. Six additional references provide supporting context but did not meet our eligibility criteria for paired biopsy-to-RP grade migration outcomes and are therefore not counted among the 37 primary studies (see below—Section 3.2).

### 3.2. Characteristics of Included Studies

The 37 included primary studies enrolled men with biopsy-diagnosed prostate adeno-carcinoma between 2004 and 2024 across North America (*n* = 11), Europe (*n* = 19), East Asia (*n* = 5), Australia (*n* = 1), and other regions (*n* = 1). Sample sizes ranged from 95 [36] to 17,598 paired biopsy–RP cases [15]. Twenty-nine studies (78%) were retrospective single-center or multicenter cohorts; three were prospective registry analyses (the UK British Association of Urological Surgeons [BAUS] Radical Prostatectomy Registry [15], the Belgian Be-RALP registry [49], and the Michigan Urological Surgery Improvement Collaborative [20]); two were prospective randomized trials (PRECISION [25] and NRG/RTOG 0126 [31]); two were prospective biomarker-driven cohorts (the NCI Trio Study [26] and the EDRN Upgrading Reference Set [41]); and one was a prospective multicenter cohort. Seven studies used the modified 2005 ISUP Gleason system as their primary framework; 27 reported their primary analysis using the 2014 ISUP GG system; and three explicitly incorporated the 2019 ISUP refinements with mandatory cribriform-pattern reporting. The biopsy approach was systematic (transrectal ultrasound [TRUS]-guided or transperineal mapping) only in 16 studies, mpMRI-targeted only or in-bore in two studies, and combined targeted-plus-systematic in 19 studies. Selected study-level characteristics and primary outcome rates are summarized in Table 1.

Following an internal eligibility review, six references were retained as supporting context citations only. The active-surveillance cohorts in this supplementary group—the Johns Hopkins Active Surveillance Program reported by Tosoian et al. (2011) [19] (*n* = 769; very-low-risk eligibility; intervention triggered by biopsy reclassification rather than paired RP grading) and its longer-term update by Tosoian et al. (2015) [57] (*n* = 1298; median 5-year follow-up; cumulative grade reclassification of 26% at 10 years and 31% at 15 years; no prostate-cancer deaths)—illustrate the magnitude and timing of biopsy-grade reclassification when RP is deferred but do not provide paired biopsy-to-RP grade outcomes because curative intervention was not the primary endpoint and most reclassified men did not proceed to immediate surgery.

Sundaresan et al. (2025) [58] reported a contemporary Yale single-institution cohort of 110 men with biopsy GG1 disease in the setting of at least one PI-RADS 5 lesion, of whom 70.5% (43/61) were reclassified to ≥GG2 on subsequent surveillance biopsy; only 15 underwent RP, with adverse pathology (≥GG3 and/or ≥pT3a) in approximately one-third, and we therefore retained this study as supporting context rather than as a primary study, since the paired biopsy-RP sample (*n* = 15) fell well below our pre-specified ≥50 paired-case threshold.

Baboudjian et al. (2025) [54], by contrast, was retained in Table 1—although it shares a primarily active-surveillance design with the cohorts above—because it uniquely provides paired biopsy-to-RP grade-migration outcomes on a defined subset: of 139 European men with GG2 disease managed with active surveillance after pre-biopsy mpMRI and image-guided biopsy across France, Spain, Italy, Switzerland, and Germany, 28 ultimately underwent RP with adverse pathology in 13 (46%) and very aggressive disease (GG ≥ 4 and/or ≥pT3b and/or pN1) in two (7%); this 28-patient RP subset reports the exact outcome of interest for our review, and we therefore included Baboudjian et al. in Table 1 with an explicit footnote indicating the 139 → 28 RP denominator, while flagging the study as supplementary because the paired case number is below our 50-case threshold.

Finally, three external-validation studies of AI-assisted Gleason grading on biopsy material—Raciti et al. (2023) [33] (Paige Prostate clinical validation across 610 needle-core whole-slide images from 218 institutions, with 18 pathologists demonstrating significant gains in sensitivity and specificity when AI-assisted relative to unassisted reading), Bulten et al. (2020) [34] (Radboud deep-learning system for Gleason grading of prostate biopsy specimens, validated against expert uropathologist reference), and Bulten et al. (2022) [35] (the PANDA challenge, a multi-institutional ensemble of crowd-sourced AI algorithms benchmarked against pathologist consensus on biopsy slides)—are cited as supporting context only because none of these reports provided a paired RP comparator and the outcome of interest in each was AI-versus-pathologist concordance at biopsy rather than biopsy-to-RP grade migration.

### 3.3. Concordance, Upgrading, and Downgrading Rates

Across the included primary studies, biopsy-to-RP grade concordance ranged from 44% to ~70%, upgrading from 14% to 67%, and downgrading from 5% to 26%. The two largest contemporary registries provided the most stable estimates: the UK BAUS Registry (*n* = 17,598; 2011–2016, modified 2005 Gleason) reported a concordance of 58.9%, upgrading of 25.5%, and downgrading of 15.6% [15], and the Belgian Be-RALP registry (*n* = 8021; robot-assisted prostatectomy era) reported a concordance of 62.9% and upgrading of 27.3% [49]. A pooled estimate from the largest age-stratified meta-analysis (27 studies, *n* = 84,296) reported an overall upgrading rate of 32.3% and an upstaging rate of 9.8% [47].

Adoption of the 2014 ISUP GG system reduced the apparent rate of clinically significant upgrading. In a four-center European cohort of 9703 RPs, simultaneous reporting using both the 2005 modified Gleason and 2014 ISUP five-tier systems demonstrated a clinically significant upgrading rate of 19.5% with the five-tier system versus 24.0% with the 2005 modified Gleason (*p* = 0.001), without a corresponding increase in downgrading (7.7% vs. 8.0%, *p* = 0.267); specificity improved from 83% to 91%, and negative predictive value from 60% to 78% [42]. The new system thus appears to better partition disease into prognostic categories without sacrificing the detection of higher-grade tumors.

Stratification by initial biopsy grade revealed that GG1 disease has the highest absolute risk of upgrading at RP. In a prospective USA multi-institutional GG1 cohort (*n* = 285) with germline whole-genome sequencing, 67% of patients were upgraded to GG ≥ 2 at RP [41]. In the Johns Hopkins active-surveillance cohort (*n* = 1298), the cumulative incidence of grade reclassification at 10 years was 26% (acknowledging that reclassification on confirmatory biopsy is methodologically distinct from upgrading at RP) [57]. Conversely, biopsy GG2 disease showed substantial heterogeneity: in a Dutch cohort of 196 men with biopsy GG2 lacking cribriform or IDC, 54.1% had an unfavorable composite endpoint at RP, defined as GG2 with cribriform/IDC at RP or any GG > 2 at RP [51], whereas in an Australian transperineal post-mpMRI cohort (*n* = 243), only 16.9% were upgraded to GG ≥ 3 [56]. This wide range likely reflects a combination of biopsy approach (transperineal post-mpMRI sampling reduces upgrading), the proportion of Gleason pattern 4 in the biopsy specimen, and undetected cribriform morphology.

#### Subgroup and Sensitivity Synthesis

Because the included studies could not be pooled quantitatively in this review (Section 2.6), between-study differences were explored through structured, pre-specified subgroup comparisons along four axes that the reviewers identified as clinically decisive: biopsy sampling strategy (systematic versus MRI-targeted or combined), imaging era (pre-MRI systematic-only cohorts versus the contemporary mpMRI-directed era), baseline risk composition (active-surveillance-eligible, predominantly GG1 cohorts versus mixed-risk cohorts), and center structure (single-center derivation cohorts versus multicenter or externally validated cohorts). A grading-system axis (modified 2005 Gleason versus 2014 ISUP GG versus 2019 ISUP refinements) was added because apparent migration rates are not directly comparable across these systems. The direction and consistency of the resulting subgroup signals are summarized in Table 2 and are carried forward into the clinical-applicability synthesis (Section 4.3.4).

Three patterns were robust across the evidence base. First, sampling strategy systematically modified migration: relative to systematic biopsy, MRI-targeted and combined sampling lowered the odds of upgrading at radical prostatectomy (combined biopsy odds ratio < 1 in the largest meta-analysis, 26 studies and 6638 patients [22]), but combined sampling approximately doubled the odds of downgrading, so the net concordance benefit depends on which form of discordance carries greater clinical cost for a given patient. Second, upgrading was concentrated in the low-grade, surveillance-eligible subgroup: biopsy GG1 carried the highest absolute upgrading risk (55–67% in dedicated cohorts [40,41]); thus, mixed-risk cohorts diluted the apparent population-level rate and partly explain between-study heterogeneity. Third, estimates derived in single-center cohorts—particularly for radiomics, machine-learning, and nomogram studies—were systematically more optimistic than the few externally validated or multicenter estimates, consistent with optimism and overfitting (Section 3.9 and Section 3.11). Grading-era effects were judged to introduce indirectness rather than a true biological signal: of the 37 primary studies, seven used the modified 2005 Gleason system, 27 reported using the 2014 ISUP GG system, and three incorporated the 2019 refinements with mandatory cribriform reporting; thus, cross-era comparisons of raw migration percentages were treated as hypothesis-generating only.

### 3.4. Clinical Predictors

**Figure 2:**
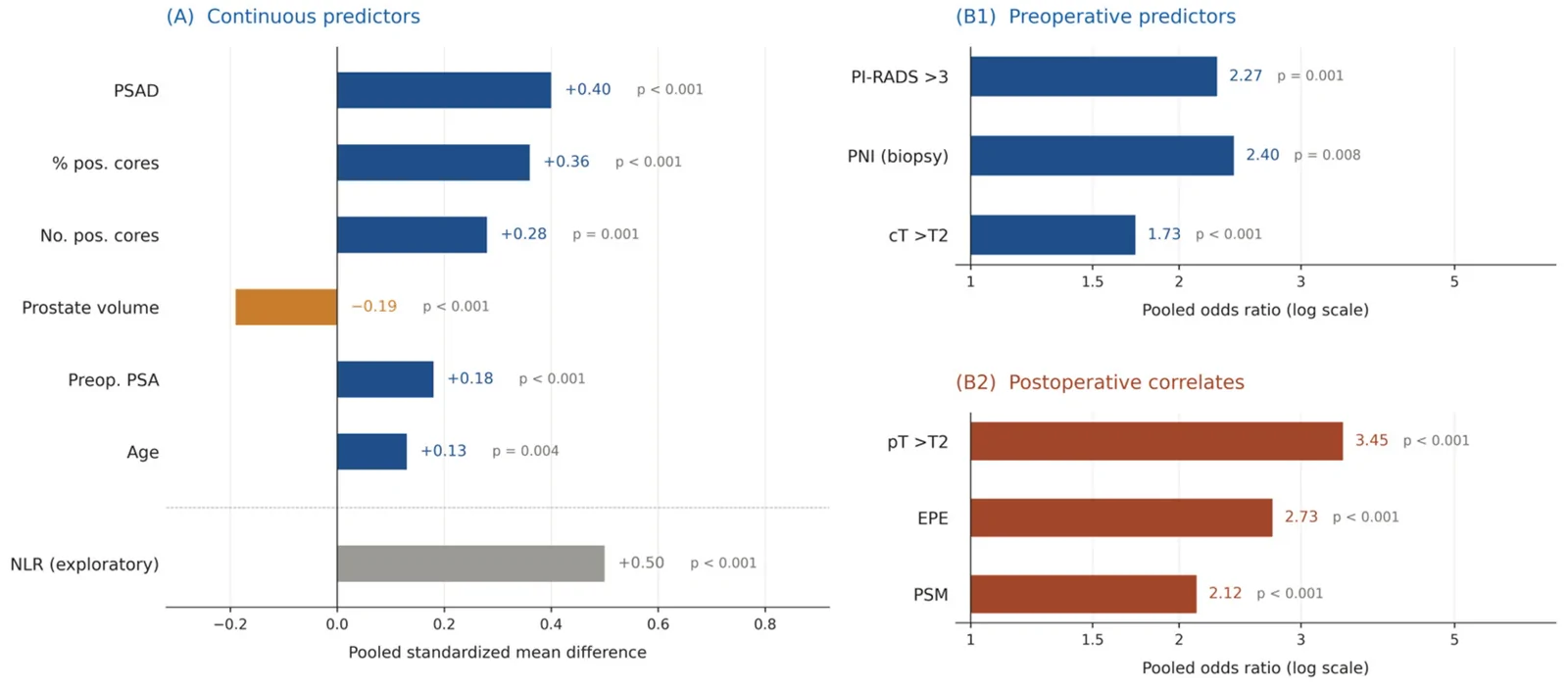
Pooled effect estimates for clinical and pathological predictors and correlates of biopsy-to-radical-prostatectomy Gleason score upgrading, derived from Wang Y et al. (2023) [37]. (**A**) Continuous clinical predictors expressed as standardized mean differences (SMDs); positive values indicate that higher predictor values are associated with upgrading, and the single negative value (prostate volume) indicates that lower volumes are associated with upgrading. The neutrophil-to-lymphocyte ratio (NLR) is plotted below a dashed separator and labelled exploratory because it associates significant inter-study publication-bias concerns and no agreed clinical threshold. (**B1**) Categorical preoperative predictors—PI-RADS category, perineural invasion (PNI) identified at biopsy, and clinical (c)T-stage—all of which are measurable at the time of biopsy and therefore meet the temporality requirement for prognostic factors under the QUIPS framework. (**B2**) Categorical postoperative pathological correlates—pathological (p)T-stage, extraprostatic extension (EPE), and positive surgical margins (PSM)—which are diagnosed at RP concurrently with the upgrading event itself. Postoperative correlates are concurrent associations rather than predictors available to the clinician at the time of biopsy and should not be incorporated into pre-treatment risk-stratification models. Pooled odds ratios for B1 and B2 are displayed on a logarithmic x-axis, with the individual reported *p*-values. Abbreviations: No. pos. cores = number of positive biopsy cores; % pos. cores = percentage of positive biopsy cores; PI-RADS = Prostate Imaging–Reporting and Data System; PSA(D) = prostate-specific antigen (density).

The Wang et al. systematic review and meta-analysis (2023) [37] is the most comprehensive synthesis of clinical predictors of biopsy-to-RP GS upgrading. Among the continuous predictors (Figure 2A), PSAD had the largest standardized effect (summary SMD 0.40, *p* < 0.001), followed by percentage of positive cores (SMD 0.36, *p* < 0.001), number of positive cores (SMD 0.28, *p* = 0.001), preoperative PSA (SMD 0.18, *p* < 0.001), and patient age (SMD 0.13, *p* = 0.004). Prostate volume was inversely associated with upgrading (SMD −0.19, *p* < 0.001). The neutrophil-to-lymphocyte ratio yielded the largest pooled SMD overall (+0.50, *p* < 0.001) but is reported separately as exploratory because of substantial inter-study publication-bias concerns and the absence of an agreed clinical cut-off [37]. Among the categorical predictors that are measurable at biopsy time (Figure 2(B1)), perineural invasion at biopsy carried a pooled OR of 2.40 (*p* = 0.008), PI-RADS category > 3 (versus ≤ 3) carried a pooled OR of 2.27 (*p* = 0.001), and clinical T-stage > T2 carried a pooled OR of 1.73 (*p* < 0.001). The same source also reports pooled odds ratios for three postoperative pathological findings (Figure 2(B2))—pathological T-stage > T2 (OR 3.45, *p* < 0.001), extraprostatic extension (OR 2.73, *p* < 0.001), and positive surgical margins (OR 2.12, *p* < 0.001)—which are reproduced for fidelity to the source but are not predictors in the prognostic-factor sense, since they are diagnosed concurrently with the radical-prostatectomy grade itself and are not available to the clinician at biopsy. An independent age-focused meta-analysis of 27 studies (*n* = 84,296) found an OR per year of age of 1.04 (95% CI 1.03–1.05) for upgrading and 1.03 (95% CI 1.01–1.04) for upstaging [47].

In the most recent prospective biopsy-to-RP cohort, Liss et al. (*n* = 285 men with GG1 disease) found that only PSAD and the percentage of cancer in positive biopsy cores remained independent predictors of upgrading on multivariable analysis [41]. Age, PSA alone, prostate volume, and family history of prostate cancer were not significant predictors in this contemporary mpMRI-era cohort, suggesting that the apparent prognostic importance of some traditional clinical variables in pooled meta-analyses may be partly explained by their correlation with sampling adequacy and by the inclusion of postoperative pathological correlates as if they were preoperative predictors.

### 3.5. Imaging Predictors

#### 3.5.1. Multiparametric MRI and PI-RADS Category

The PI-RADS category emerged as the most reproducible imaging predictor of biopsy-to-RP upgrading. The Wang Y et al. meta-analysis [37] reported a pooled OR of 2.27 (*p* = 0.001) for PI-RADS > 3 versus ≤ 3. Among individual studies, the strongest signal was reported by Dekalo et al. in a 127-patient cohort of biopsy ISUP 1 disease, where PI-RADS 4–5 was associated with upgrading at RP, with an adjusted OR of 24.3 (95% CI 7.3–80.5) compared with equivocal or non-suspicious findings; 84% of PI-RADS 4–5 patients were upgraded versus 26% of others (*p* < 0.001) [59]. In a contemporary cohort of biopsy GG1 patients with at least one PI-RADS 5 lesion (*n* = 110), 70.5% were reclassified on follow-up biopsy, and one-third of those proceeding to RP showed adverse pathology (≥GG3 and/or ≥pT3a) [58]. In biopsy GG2 disease without cribriform/intraductal pattern, PI-RADS 5 was independently associated with upgrading (OR 2.17, 95% CI 1.03–4.70) [51]; the percentage of Gleason pattern 4 (OR 1.54 per 10% increase, 95% CI 1.17–2.07) and cT3 stage (OR 3.60, 95% CI 1.08–14.50) were similarly predictive.

#### 3.5.2. MRI Radiomics and Machine-Learning Models

Five included studies developed MRI-radiomics or MLMs specifically to predict biopsy-to-RP upgrading (Zhang et al. 2020 [60]; Marvaso et al. 2024 [61]; Ozbozduman et al. 2024 [36]; Wang G et al. 2023 [62]; and Soeterik et al. 2024 [63]); together with three clinical nomograms (Wang X et al. 2021 [50]; Karamık et al. 2024 [64]; and Cano Garcia et al. 2023 [65]), these constitute the eight prediction-model studies evaluated under PROBAST (Table 2, Table 3). The largest single-institution model (Zhang et al., *n* = 166) combined T2-weighted, ADC, and dynamic contrast-enhanced features with clinical variables and achieved an AUC of 0.910 in both training and validation cohorts, with adequate calibration (Hosmer–Lemeshow *p* = 0.624 and 0.294, respectively) [60]. A 2024 gradient-boosted MLM trained on 949 patients reached AUCs of 0.73–0.96 across six pathology endpoints, with whole-prostate radiomics adding a small but measurable boost over clinical variables alone [61]. Apparent diffusion coefficient (ADC) features alone reached AUCs of 0.71–0.81 in smaller cohorts. Across all radiomics studies, external validation was performed in only one of eight, and methodological reporting following the TRIPOD+AI statement was inconsistent.

#### 3.5.3. PSMA-PET/CT

The Chen DC et al. systematic review and meta-analysis (2025) of 23 studies of PSMA-PET demonstrated that intraprostatic SUV_max_ increases monotonically with ISUP GG and pathological tumor stage [28]. Pooled SUV_max_ rose from 5.8 (95% CI 3.9–7.7) for RP ISUP 1 disease to 17.3 (95% CI 13.1–21.5) for ISUP 5; from 9.7 (95% CI 7.8–11.5) for pT2 to 13.8 (95% CI 10.9–16.7) for pT3/4. However, despite the clear gradient in pooled point estimates, substantial inter-study heterogeneity (I^2^ > 50%) was observed across all subgroups. This reported heterogeneity is consistent with the methodological dispersion documented: contributing studies used three different radiotracer chemistries ([^68^Ga]Ga-PSMA-11, [^68^Ga]Ga-PSMA-I&T, and the ^18^F-labelled agents [^18^F]PSMA-1007 and [^18^F]DCFPyL), a mixture of weight-based and fixed-dose administration protocols (e.g., 1.25–4.0 MBq/kg vs. fixed doses ranging from approximately 100 to 200 MBq), uptake intervals between tracer injection and image acquisition spanning from 40–60 min to over 2 h, and inconsistent volume-of-interest definitions (single-voxel SUV_max_ vs. 40–41% isocontour thresholds within a 3D VOI vs. fixed-threshold SUV > 4 segmentation). Because each of these factors directly affects measured SUV_max_ independently of underlying tumor PSMA expression, single-threshold SUV_max_ cut-offs derived from the pooled estimate are unlikely to be transportable across centers without local recalibration to the specific tracer, dose, uptake window, and segmentation protocol in use [28]. Indeed, Chen et al. cautioned explicitly against using a single SUV_max_ threshold for individual-patient decisions in clinical practice [28].

**Figure 3:**
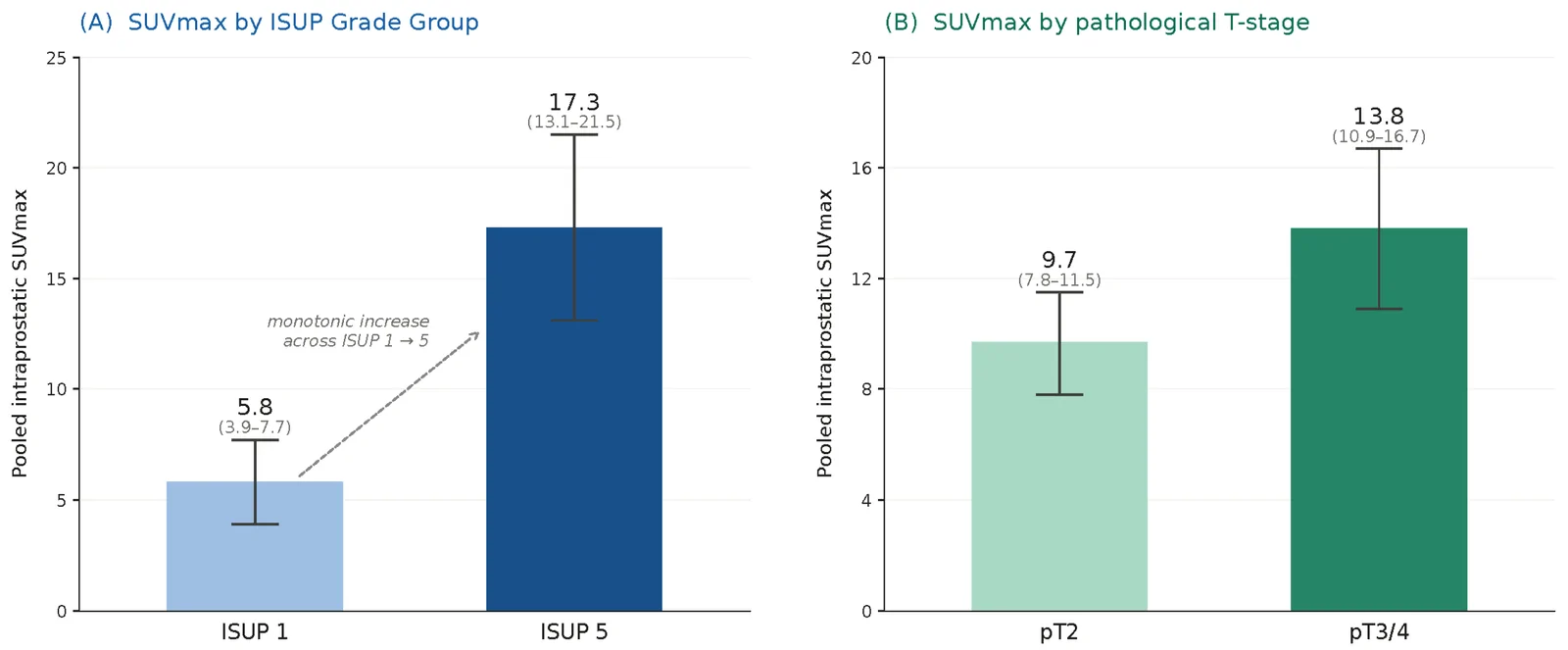
Pooled intraprostatic PSMA-PET maximum standardized uptake value (SUV_max_) stratified by RP histopathology—derived from the systematic review and random-effects meta-analysis of 23 studies by Chen DC et al. (2025) [28]: (**A**) Pooled SUV_max_ by ISUP Grade Group at RP—between the plotted boundary pooled values for ISUP 1 and ISUP 5, a monotonic increase occurs across the intermediate ISUP 2, 3, and 4 strata, indicated by the dashed arrow; (**B**) Pooled SUV_max_ by pT-stage. Bars represent the pooled point estimate; whiskers indicate the 95% CI reported. Substantial inter-study heterogeneity (I^2^ > 50%) was observed across all subgroups, which explicitly discourages the use of single SUV_max_ thresholds for clinical decision-making in individual patients. Abbreviations: CI = confidence interval; ISUP = International Society of Urological Pathology; pT2 = pathological T2 stage (organ-confined disease); pT3/4 = pathological T3 (extraprostatic extension) or T4 stage (invasion of adjacent structures); RP = radical prostatectomy.

A complementary pooled analysis specifically addressing pathological upgrading reported a sensitivity of 0.68 (95% CI 0.60–0.76) and specificity of 0.74 (95% CI 0.59–0.85) [48]. In a multicenter international cohort of 605 patients, PSMA-derived intraprostatic tumor volume ≥ 2 vs. <2 carried an adjusted OR of 6.36 (95% CI 1.47–27.6) for upgrading to GG ≥ 4 in patients with biopsy GG1–3 disease [63]. Pooled SUV_max_ values stratified by RP histopathology rose from 5.8 (95% CI 3.9–7.7) for RP ISUP 1 disease to 17.3 (95% CI 13.1–21.5) for ISUP 5 (Figure 3A); from 9.7 (95% CI 7.8–11.5) for pT2 to 13.8 (95% CI 10.9–16.7) for pT3/4 (Figure 3B).

### 3.6. Biopsy Approach: Systematic, MRI-Targeted, Combined; Transrectal vs. Transperineal; In-Bore

**Figure 4:**
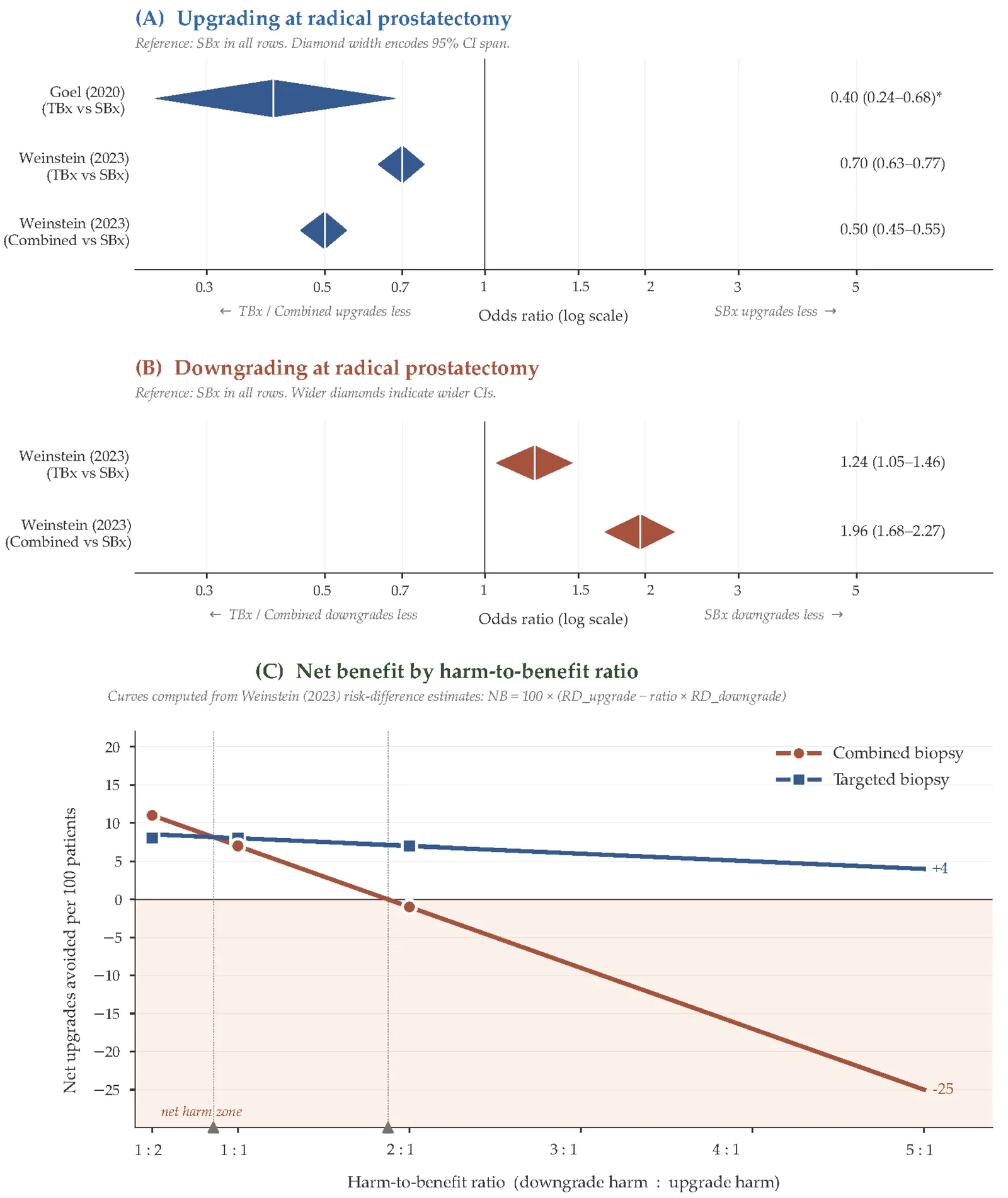
Effect of biopsy approach on biopsy-to-RP ISUP grade group migration. (**A**) Pooled odds ratios (ORs) for upgrading at RP—adapted from Goel et al. (2020) [27] and Weinstein et al. (2023) [22]; all rows use SBx as the reference. Goel’s published OR (2.47, 95% CI 1.48–4.14) has been inverted (1/OR; CI bounds reversed) to 0.40 (95% CI 0.24–0.68) for direct comparability with Weinstein’s convention and is marked with an asterisk (*). Diamond width encodes 95% CI span, not study weight. (**B**) Pooled ORs for downgrading at RP [22]; both TBx and combined biopsies result in more downgrading than SBx alone, with the effect greatest for combined biopsy. (**C**) Net-benefit decision curves—computed from the published risk-difference estimates [22]. Markers indicate the three explicitly reported points (1:2, 1:1, 2:1); curves extend the same formula to 5:1. Combined biopsy yields greater net benefit than TBx below a harm-to-benefit ratio of ~0.86:1 and crosses into net harm above ~1.88:1, while TBx retains positive net benefit across the full range. Abbreviations: CI = confidence interval; OR = odds ratio; RD = risk difference; RP = radical prostatectomy; SBx = systematic transrectal-ultrasound-guided biopsy; TBx = MRI-targeted biopsy.

The Goel et al. (2020) [27] and Weinstein et al. (2023) [22] systematic reviews and meta-analyses provide the most rigorous comparative evidence on biopsy approach. Goel and colleagues [27], pooling 8 of 10 included studies in the upgrading analysis (full meta-analytic cohort *n* = 1215 men) through 2018, showed that systematic biopsy was significantly more likely to upgrade at RP than MRI-targeted biopsy (OR 2.47, 95% CI 1.48–4.14, *p* = 0.001) without an offsetting increase in downgrading (OR 1.13, 95% CI 0.48–2.67, *p* = 0.783). Weinstein et al. [22], extending this analysis to 26 studies and 6638 patients through 2022, additionally distinguished combined biopsy from targeted-only biopsy: targeted biopsy reduced the odds of upgrading versus systematic (OR 0.70, 95% CI 0.63–0.77; *p* < 0.001), and combined biopsy reduced them further (OR 0.50, 95% CI 0.45–0.55; *p* < 0.001), as shown in Figure 4A. However, combined biopsy approximately doubled the odds of downgrading at RP (OR 1.96, 95% CI 1.68–2.27; *p* < 0.001; Figure 4B), and on formal net-benefit decision-curve analysis (Figure 4C), the apparent advantage of combined biopsy reversed under realistic patient-preference weights: the net gain in concordance was +8 versus +7 per 100 men when upgrading and downgrading harms were weighted equally, flipped to +7 versus −1 per 100 when downgrading was weighted twice as harmful as upgrading, and combined biopsy crossed into net harm at any harm-to-benefit ratio above approximately 1.88:1. Targeted biopsy, by contrast, retained positive net benefit across the full reported range (harm-to-benefit ratios from 1:2 to 5:1). Because the two meta-analyses pool different study sets and time windows, modest numeric divergence between Goel’s and Weinstein’s targeted-versus-systematic estimates is expected; both nevertheless support the same qualitative conclusion that MRI-targeted biopsy reduces upgrading at RP compared with systematic biopsy alone.

In the Trio Study (NEJM, *n* = 404 RPs nested within 2103 biopsied men), combined biopsy yielded the lowest upgrading rate at RP (14.4%), followed by MRI-targeted alone (30.9%) and systematic alone (41.6%). Upgrading to GG ≥ 3 followed the same pattern: combined 3.5%, targeted 8.7%, and systematic 16.8% [26].

Comparisons of transrectal versus transperineal biopsy consistently favored the transperineal route in the post-mpMRI era. Hagens et al. (Netherlands, *n* = 1058) reported that transperineal biopsy was independently associated with biopsy–RP concordance (OR 1.33, 95% CI 1.01–1.75, *p* = 0.04) and reduced AUA risk-group migration (OR 0.70, 95% CI 0.52–0.93, *p* = 0.01) [52]. Zattoni et al., for the EAU-YAU Prostate Cancer Working Group (*n* = 1282), likewise found that transperineal MRI-targeted biopsy increased concordance versus the transrectal approach in multivariable analysis [53]. A Polish single-center study reported a paired biopsy–RP subset of 68 men who underwent transperineal combined fusion biopsy (ComBx) and 89 men who underwent standard TRUS-guided biopsy (TRUS-Bx), drawn from a larger pool of 250 ComBx and 250 TRUS-Bx procedures performed at the index institution. In the paired subset, concordance was 63% (ComBx) versus 49% (TRUS-Bx) (*p* = 0.042) and upgrading was 16% versus 35% (*p* = 0.004) [55]. In-bore MRI-guided biopsy reached intermediate concordance values (64.2% in the largest single-center series, *n* = 95 [36]) and may further reduce upgrading when combined with MLMs, although prospective head-to-head data with combined MRI-fusion biopsy remain limited.

### 3.7. Cribriform and Intraductal Carcinoma at Biopsy

Cribriform pattern and IDC are independently prognostic but are systematically under-detected on biopsy. Two included studies that explicitly evaluated biopsy sensitivity for these features against RP whole-mount pathology reported sensitivities of 34–57% and specificities of 87–95%. Masoomian et al. (*n* = 245) reported a biopsy sensitivity of 47.2% and specificity of 94.9%; biopsy detection of cribriform/IDC was independently associated with advanced pathological stage (*p* = 0.013) [66]. Ericson et al. (*n* = 455) reported a biopsy sensitivity of 56.5% overall but only 34.1% in active-surveillance-eligible patients, with a specificity of 87.2%; the addition of mpMRI fusion biopsy did not improve sensitivity (53.5%) [67]. In the largest cohort, Bernardino et al. (*n* = 836) reported that undetected cribriform/IDC at biopsy was independently associated with biochemical recurrence after RP (adjusted HR 2.14, 95% CI 1.41–3.25, *p* < 0.001) [68]. The implication is that approximately half of all men with cribriform/IDC at RP have a falsely reassuring biopsy and that this subgroup is the principal driver of clinically significant biopsy-to-RP upgrading.

### 3.8. Genomic and Biomarker Tests

#### 3.8.1. Decipher and Other Tissue-Based Genomic Classifiers

The Decipher 22-gene genomic classifier was evaluated in three included reports. Press et al. analyzed an active-surveillance cohort (*n* = 133) and reported that the biopsy Decipher score was associated with subsequent biopsy Gleason upgrading on univariable analysis (OR 1.24 per 0.10-unit increase; *p* = 0.045), with similar effect sizes in the GG1 stratum [29]. On further multivariable analysis adjusting for age, baseline PI-RADS, PSAD, and number of positive systematic biopsy cores, the Decipher score remained independently associated with biopsy upgrading (OR 1.37 per 0.10-unit increase, 95% CI 1.05–1.79; *p* = 0.02) [29]. The MUSIC confirmatory-testing pathway integrated Decipher and Prolaris (cell-cycle progression score) alongside confirmatory MRI to refine active-surveillance candidacy and reduce subsequent upgrading at RP [20]. The NRG Oncology Decipher analysis confirmed prognostic performance across intermediate-risk strata [31].

#### 3.8.2. Prostate Health Index, 4Kscore, and Urinary Biomarkers

The PHI was evaluated in three included primary studies. Yan et al. (*n* = 351) reported that the PHI independently predicted upgrading from biopsy GG ≤ 2 to RP GG ≥ 3 in low-risk patients with an OR of 1.80 (95% CI 1.14–2.82, *p* = 0.01) and that the related PHI density (PHID) had an OR of 2.34 (95% CI 1.30–4.20, *p* = 0.005); both metrics improved AUC from 0.59 (base clinical model) to 0.69 (PHI) and 0.71 (PHID) [69]. Maxeiner and colleagues (*n* = 437 RPs) found that the PHI was independently associated with adverse RP pathology (≥pT3, OR 2.20; GS ≥ 7, OR 2.09); a PHI cut-off of 46.4 was identified [70]. Kim et al. (*n* = 71 RPs) confirmed that PHI was an independent predictor of GS upgrading and adverse RP pathology on multivariable analysis [30]. Biomarker panels combining serum or urinary assays with mpMRI have been explored in early-phase studies for predicting high-grade disease at biopsy, but contemporary peer-reviewed evidence specifically reporting paired biopsy-to-RP grade migration outcomes for the 4Kscore, ExoDx Prostate (IntelliScore), or SelectMDx assays in MRI-era cohorts is not yet available. Separately, a pre-specified four-kallikrein post-radical-prostatectomy model—derived from a Memorial Sloan Kettering–Hamburg cohort of 2330 men—was significantly associated with adverse surgical pathology (OR 1.49, 95% CI 1.32–1.67) and biochemical recurrence (HR 1.16, 95% CI 1.06–1.26), with the strongest discrimination in biopsy GG3 + 3 disease [71].

#### 3.8.3. Germline Mutations

The largest prospective evaluation of germline mutations as predictors of biopsy-to-RP grade migration was performed by Liss et al. (*n* = 285 men with biopsy GG1, of whom 192 had germline whole-genome sequencing) [41]. Despite a 67% overall upgrading rate to RP GG ≥ 2, no germline factor—neither pathogenic variants in DNA-damage-repair genes (*BRCA1*, *BRCA2*, *ATM*, *CHEK2*, *MSH2*, *HOXB13*) nor a polygenic risk score for prostate cancer—was a significant predictor of upgrading. Only PSAD and the percentage of cancer in positive biopsy cores remained significant on multivariable analysis. The current direct evidence therefore does not support germline status as an independent predictor of biopsy-to-RP upgrading once the initial biopsy grade is known, although prior studies have shown that pathogenic germline mutations in DNA-damage-repair genes are enriched in high-grade prostate cancer at diagnosis [32].

### 3.9. Artificial-Intelligence and Digital-Pathology Models

Two included studies evaluated AI systems for tasks relevant to biopsy-to-RP grade migration; three additional reports of AI-assisted Gleason grading on biopsy whole-slide images (Radboud [34], PANDA [35], Paige Prostate [33]) did not provide paired biopsy-to-RP grade-migration outcomes meeting the eligibility criteria and are cited as supporting context only. Two distinct paradigms remain visible in the AI literature.

The first paradigm comprises AI-assisted Gleason grading on biopsy whole-slide images, which aims to reduce inter-pathologist variability at the source. The Radboud deep-learning system [34] achieved a quadratic-weighted κ of 0.62 against the expert reference, within the range of inter-pathologist agreement; the PANDA challenge consortium [35] subsequently demonstrated that an ensemble of crowd-sourced algorithms reached pathologist-level performance on multi-continental external validation. The Paige Prostate platform, the first FDA-cleared AI prostate pathology product [33], demonstrated improved sensitivity and specificity in pathologist-assisted reading and in a 55-patient pilot, improved AI-assisted Gleason concordance with reference RP pathology by 13% [33].

The second paradigm comprises predictive models using clinical and biopsy MRI features to forecast RP grade. Ozbozduman et al. trained an MLM on data from 95 in-bore MRI-guided biopsies and reached an accuracy of 85.6% for predicting upgrading at RP, compared with 64.2% concordance for biopsy GG alone [36]. A single-center Chinese tree-augmented naïve Bayesian network model trained on transperineal combined biopsies (*n* = 356) and internally validated by a 70/30 split-sample reported an apparent AUC of 0.81 and accuracy of 77%, identifying lymphatic metastasis, number of positive biopsy cores, ISUP stage, and PI-RADS score as the top predictors of upgrading [62]. None of the included MLMs has yet undergone prospective external validation in a randomized comparison against standard reading; reporting against the TRIPOD+AI statement was incomplete in both studies.

Judged against the methodological standards that determine whether a prediction model is trustworthy, the AI and radiomics literature in this field is still early-phase, which is the explicit basis for its low-to-very-low GRADE certainty. Five noteworthy weaknesses recur across studies. First, training/testing separation is frequently inadequate: the strongest reported discrimination came from single-institution models with internal validation only (for example, the Zhang radiomics model apparent AUC of 0.910 in both training and an internal validation split [60]; the Karamık U.P.G.R.A.D.E. score and apparent AUC of 0.952 with split-sample validation only [64]), and apparent AUCs of this magnitude in development cohorts of 95–356 patients are very likely optimistic estimates of out-of-sample performance. Second, calibration—whether predicted probabilities match observed frequencies, which matters far more than discrimination for individual counselling—was reported in only a minority of studies (Zhang reported Hosmer–Lemeshow statistics [60]; most others reported none); thus, a model could discriminate well yet systematically over- or under-state a given man’s upgrading probability. Third, explainability is limited: tree-augmented Bayesian and gradient-boosted models [61,62] do rank their inputs (e.g., positive-core number, PI-RADS, PSAD), but the radiomics-feature models offer little interpretable mapping from voxel-level texture features to biology, which constrains clinical trust and regulatory acceptance. Fourth, reproducibility pipelines are usually unreported: image acquisition parameters, segmentation method, feature-extraction software and version, harmonization across scanners, and random seeds were rarely specified completely, so independent re-implementation is generally not possible from the published text. Fifth, multicenter external validation is essentially absent: across the eight prediction-model studies, only one (the Cano Garcia SEER-based downgrading nomogram [65]) was externally validated in an unaffiliated cohort, and none of the five MRI-radiomics/machine-learning models was; small development cohorts drawn from single referral centers additionally raise the risk of overfitting, feature-selection bias (where the same data inform feature selection and performance estimation), and spectrum bias. Taken together, these limitations place AI- and radiomics-based predictors of biopsy-to-RP migration as exploratory tools suited to research and multidisciplinary-team contexts rather than instruments ready for routine standalone clinical decision-making.

### 3.10. Multivariable Prediction Models

Eight included studies reported the development or validation of a multivariable prediction model for biopsy-to-RP grade migration. Discrimination, as measured by AUC or C-index, ranged from 0.726 (Wang nomogram, internally-validated in a Chinese cohort, *n* = 198 [50]) to 0.952 (the U.P.G.R.A.D.E. score by Karamık et al., *n* = 235 [64]). This apparent AUC was internally split-sample-validated only, not bias-corrected by bootstrap or externally validated, and should therefore be regarded as a development-cohort upper bound likely to over-estimate out-of-sample discrimination (Section 4.4). Seven of the eight models reported only internal validation (bootstrap or split-sample); only one model (Cano Garcia et al.’s SEER-based downgrading nomogram [65]) underwent independent external validation in an unaffiliated cohort. The most parsimonious models combined PSA, PSAD, the percentage of positive biopsy cores, and PI-RADS category, augmented in the more recent models by mpMRI-derived radiomics or genomic-classifier scores. No included model combined external validation in an unaffiliated cohort, complete reporting of calibration metrics (intercept, slope, and Hosmer–Lemeshow), and adherence to the TRIPOD+AI statement; on this combined criterion, no current model meets the threshold for unmodified clinical deployment. Selected prediction models are summarized in Table 3.

### 3.11. Risk of Bias in Included Studies

Risk of bias was assessed using QUIPS for prognostic-factor studies, PROBAST for prediction-model studies, and QUADAS-2 for diagnostic-accuracy studies. A summary appears in Table 4. The most common QUIPS concerns were in the domains of study confounding (potential residual confounding by un-reported biopsy approach, mpMRI use, or pathology re-review) and prognostic-factor measurement (heterogeneity in the definition and reporting of cribriform/IDC, percentage of Gleason pattern 4, and PI-RADS version). PROBAST flagged the analysis domain as high risk of bias in five of eight prediction-model studies, primarily because of insufficient sample size, inadequate handling of missing data, or absent external validation. QUADAS-2 concerns in studies of mpMRI and PSMA-PET against RP whole-mount pathology centered on the index-test domain (variability in PI-RADS version and PET tracer) and patient-selection domain (over-representation of higher-risk cohorts undergoing both modalities).

**Figure 5:**
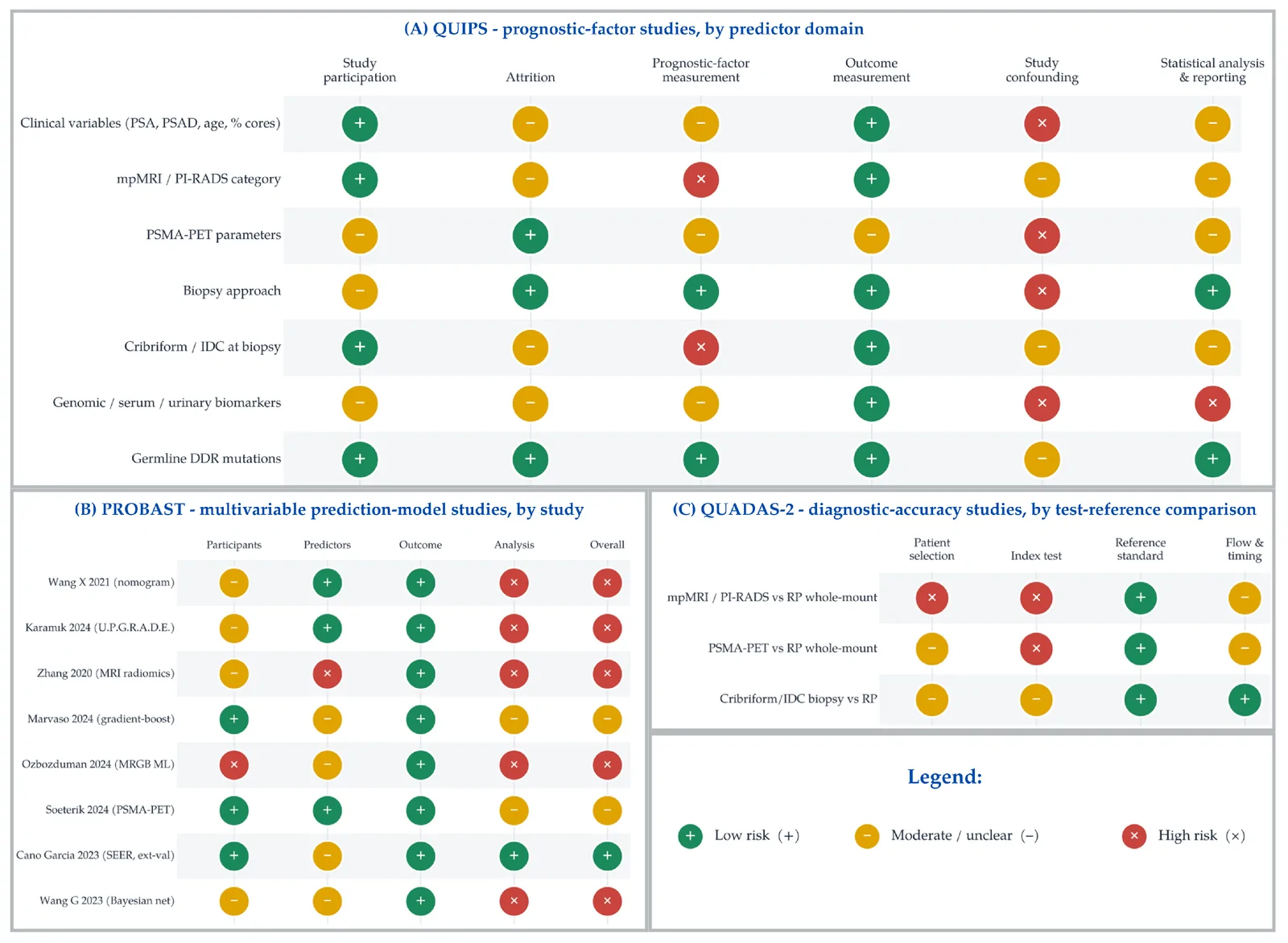
Domain-level risk-of-bias (“traffic-light”) summary across the included studies, presented separately for each appraisal instrument. (**A**) QUIPS judgements for prognostic-factor studies, grouped by predictor domain; the six columns correspond to the QUIPS domains of study participation, attrition, prognostic-factor measurement, outcome measurement, study confounding, and statistical analysis and reporting. (**B**) PROBAST judgements for the eight multivariable prediction-model studies, shown per study across the Participants, Predictors, Outcome, and Analysis domains together with the overall judgement. The eight models shown are Ozbozduman et al. 2024 [36], Wang X et al. 2021 [50], Zhang et al. 2020 [60], Marvaso et al. 2024 [61], Soeterik et al. 2024 [63], Wang G et al. 2023 [62], Karamık et al. 2024 [64], and Cano Garcia et al. 2023 [65]. (**C**) QUADAS-2 judgements for diagnostic-accuracy comparisons of an index test against radical-prostatectomy whole-mount pathology, across the patient-selection, index-test, reference-standard, and flow-and-timing domains. Each circle encodes the domain-level rating: green with “+” = low risk of bias, amber with “−” = moderate or unclear risk, and red with “×” = high risk. Study confounding (QUIPS), the analysis domain (PROBAST), and patient selection and the index test (QUADAS-2) were the domains most frequently rated at high risk; the corresponding signaling-question-level judgements are recorded in the data-extraction file. Abbreviations: IDC = intraductal carcinoma; DDR = DNA damage repair; mpMRI = multiparametric magnetic resonance imaging; MRGB = MRI-guided biopsy; ML = machine learning; PI-RADS = Prostate Imaging–Reporting and Data System; PSA = prostate-specific antigen; PSAD = PSA density; PSMA-PET = prostate-specific membrane antigen positron emission tomography; QUADAS-2 = Quality Assessment of Diagnostic Accuracy Studies, version 2; QUIPS = Quality In Prognosis Studies; PROBAST = Prediction model Risk Of Bias Assessment Tool; RP = radical prostatectomy; SEER = Surveillance, Epidemiology, and End Results; U.P.G.R.A.D.E. = published prediction-score name (Karamık et al. 2024 [64]).

A domain-level “traffic-light” visualization of these judgements, separated by instrument (QUIPS, PROBAST, and QUADAS-2), is provided below (Figure 5), allowing the pattern of bias across domains to be read at a glance. Three domain-level patterns dominate. In the prognostic-factor (QUIPS) studies, study confounding is the most frequent high-risk domain—few cohorts adjusted simultaneously for biopsy approach, mpMRI use, pathology re-review, and case-mix—followed by prognostic-factor measurement, reflecting inconsistent definition and reporting of cribriform/IDC, percentage of Gleason pattern 4, and PI-RADS version. In the diagnostic-accuracy (QUADAS-2) studies, patient selection and the index-test domain carry the most concern, because cohorts undergoing both advanced imaging and RP are enriched for higher-risk disease (spectrum effects) and because PI-RADS version and PET tracer/protocol varied between studies. In the prediction-model (PROBAST) studies, the analysis domain drives the overall high-risk rating in five of eight models.

For the AI/radiomics subset specifically, the analysis-domain concern resolves into four interrelated, well-recognized failure modes that weighed heavily in the certainty rating: (i) overfitting, expected when flexible models with many candidate radiomic features are trained on development cohorts of fewer than a few hundred patients; (ii) the absence of external validation, since none of the five MRI-radiomics/machine-learning models was validated in an independent cohort; (iii) the small effective sample size relative to the number of candidate predictors, breaching the events-per-variable conditions assumed by PROBAST; and (iv) feature-selection bias, when feature selection is performed on the same data used to estimate performance and is not nested within resampling. These four issues, together with incomplete TRIPOD+AI reporting, account for the lowest certainty rating, assigned to the machine-learning/radiomics row in Table 5 (Section 3.12).

### 3.12. Certainty of Evidence (GRADE)

Certainty of evidence (GRADE) was rated as moderate for PI-RADS category, biopsy approach (combined vs. systematic), PSMA-PET SUV_max_ (downgraded once for inconsistency), and PSAD; low for cribriform/IDC at biopsy (downgraded for risk of bias and indirectness related to inconsistent reporting), the Decipher genomic classifier, the PHI, and MLMs for biopsy-to-RP migration; and very low for germline mutations as direct predictors of biopsy-to-RP upgrading (downgraded for imprecision and indirectness, given that the only large prospective study was negative). A consolidated GRADE summary-of-findings table is provided as Table 5.

## 4. Discussion

### 4.1. Principal Findings

This systematic review synthesized 37 contemporary primary studies and eight prior systematic reviews and meta-analyses [13,16,22,27,28,37,47,48] addressing biopsy-to-RP ISUP GG migration and its predictors. Three principal findings emerge. First, despite a decade of refinements in grading (the 2014 and 2019 ISUP consensus conferences), in imaging (mpMRI with PI-RADS reporting and PSMA-PET), and in biopsy technique (MRI-targeted, combined, and transperineal sampling), biopsy-to-RP grade discordance remains substantial: across our included studies, concordance ranged from 44% to ~70%, upgrading from 14% to 67%, and downgrading from 5% to 26%, with the two largest contemporary registries—the UK BAUS Registry (*n* = 17,598) and the Belgian Be-RALP registry (*n* = 8021)—anchoring concordance at approximately 59–63% and upgrading at 25–27% [15,49]. The 2014 five-tier GG system did reduce the apparent rate of clinically significant upgrading from 24.0% to 19.5% in a 9703-RP European cohort without an offsetting rise in downgrading [42], but the underlying biological and sampling problem is not solved by reclassification alone.

Second, biopsy GG1 disease—the population for whom misclassification carries the greatest active surveillance consequences—has the highest absolute risk of upgrading at RP: 67% in a contemporary USA prospective cohort with germline whole-genome sequencing [41] and a 26% cumulative incidence of grade reclassification at 10 years in the Johns Hopkins active-surveillance cohort (*n* = 1298) [57]. Biopsy GG2 disease shows wide between-cohort variability (16.9% upgrading to ≥GG3 in an Australian transperineal post-mpMRI cohort [56] versus 54.1% with an unfavorable composite endpoint at RP (GG2 with cribriform/IDC, or any GG > 2) in a Dutch cohort of biopsy GG2 patients without baseline cribriform/IDC [51], confirming that sampling adequacy and undetected aggressive morphology, rather than intrinsic GG2 biology alone, dominate the discordance signal.

Third, of the emerging predictors examined, only PI-RADS category, biopsy approach (combined versus systematic), PSMA-PET SUV_max_, and PSAD reached moderate GRADE certainty in our synthesis; cribriform/IDC at biopsy, the Decipher genomic classifier, the PHI, and MLMs were rated low certainty; and germline mutations as direct predictors of biopsy-to-RP upgrading were rated very low certainty (the only large prospective evaluation was negative [41]). No predictor reached high certainty. The combined PROBAST and GRADE picture sets a clear ceiling on what can currently be claimed. None of the five MRI-radiomics or machine-learning model studies was externally validated, and all five were rated high risk of bias on the PROBAST analysis domain, supporting very low GRADE certainty. Among clinical nomograms, only the Cano Garcia et al. (2023) external-validation cohort demonstrated adequate external validity (AUC 0.72 for any downgrading [65]), supporting low certainty for that subgroup.

### 4.2. Comparison with Prior Studies

Our findings extend, and in places revise, prior syntheses. The 2008 Cohen meta-analysis reported a biopsy-to-RP concordance of 63%, with 30% upgrading and 7% downgrading [13]; the 2012 Hopkins series of 7643 paired specimens showed 36.3% upgrading from biopsy GS5–6 to ≥GS7 [14]. Two patterns are evident in the contemporary data assembled here. The proportion of upgrading has fallen modestly with mpMRI-targeted and combined biopsy adoption—most clearly in the Trio Study, where combined biopsy yielded 14.4% upgrading versus 30.9% for targeted alone and 41.6% for systematic alone [26]—but downgrading has correspondingly risen (15.6% in BAUS [15], 15% in the Anser collaborative [21]), driven by the same targeted-sampling change.

Two recent Anser-cohort analyses extend this further by demonstrating that targeted biopsies, particularly in smaller tumors, may now systematically overestimate ISUP GG [21]—a direction-of-bias finding that was not visible in the systematic-biopsy era and that complicates the simple narrative that more sensitive sampling necessarily yields a more accurate grade. The 2025 Wu et al. meta-analysis of 48 studies and 63,119 biopsy GG2 patients reported median (IQR) upgrading to ≥GG3 of 23.4% and to ≥GG4 of 3.6% [16], consistent with the GG2-stratified rates seen across our included studies.

For biopsy approach, our synthesis confirms the pooled estimates of Goel et al. (systematic versus targeted: OR for upgrading 2.47, 95% CI 1.48–4.14) [27] and of Weinstein et al. (targeted versus systematic OR 0.70; combined versus systematic OR 0.50; combined versus systematic OR for downgrading 1.96) [22] but adds a clinically important reframing: Weinstein’s formal net-benefit analysis showed that the gain in concordance was 8 versus 7 per 100 men if upgrading and downgrading harms were weighted equally but flipped to 7 versus −1 per 100 if downgrading harm was weighted more heavily, indicating that combined biopsy may carry net harm under realistic patient-preference weights. Our review is the first, to our knowledge, to integrate this net-benefit reframing alongside imaging, molecular, and AI-based predictors under one PRISMA 2020-compliant framework. By contrast, the Wang Y et al. meta-analysis of clinical predictors [37] and the Chen DC et al. PSMA-PET meta-analysis [28] each addressed a single predictor domain; the present work synthesizes evidence across imaging, molecular, and AI predictors and contextualizes each within the same risk-of-bias and certainty framework.

### 4.3. Clinical Implications

Beyond quantifying the extent of discordance, a central translational question is how the differing certainty of established and emerging predictors should shape contemporary practice. The PI-RADS category, biopsy approach, PSA density, and PSMA-PET SUV_max_—the four predictors reaching moderate GRADE certainty in this review—are already actionable and can inform risk stratification at the point of biopsy: they help identify which biopsy GG1–GG2 patients merit confirmatory imaging or resampling before an active-surveillance decision is finalized, and they inform treatment-planning discussions about nerve sparing and the extent of lymph node dissection. Genomic classifiers, radiomic signatures, and AI-based models, by contrast, remain of low-to-very-low certainty and are not yet positioned to independently redirect these decisions; at present, they are best used as supportive information within a multidisciplinary discussion rather than as standalone triggers. The following sections examine how this actionable-versus-investigational distinction applies to active surveillance selection (Section 4.3.1), treatment selection (Section 4.3.2), and biopsy strategy (Section 4.3.3), before Section 4.3.4 formalizes the distinction as an evidence-tiered taxonomy.

#### 4.3.1. Active Surveillance

The 67% upgrading rate observed in the Liss prospective biopsy GG1 cohort [41] and the finding that, in a contemporary cohort of 110 biopsy GG1 patients with at least one PI-RADS 5 lesion, 70.5% (43/61) of those who underwent additional biopsy were reclassified [58] together argue that contemporary active surveillance protocols cannot rely on biopsy GG alone. The cribriform/IDC sensitivity gap is a particular concern in this setting: Ericson et al. reported a biopsy sensitivity of only 34.1% for cribriform pattern in active-surveillance-eligible patients [67], and Bernardino et al. reported that undetected cribriform/IDC at biopsy was independently associated with biochemical recurrence after RP (adjusted HR 2.14, 95% CI 1.41–3.25) [68]. Confirmatory testing pathways, such as the Michigan Urological Surgery Improvement Collaborative (MUSIC), which integrate confirmatory mpMRI and selective genomic-classifier testing [20], are a rational response to this evidence—although the negative germline-predictor finding from Liss [41] indicates that current germline panels do not, by themselves, refine biopsy-GG1-based AS eligibility.

A question of direct relevance to surveillance counselling is whether men on active surveillance who are later upgraded at surgery carry a higher risk of dying from prostate cancer. The present evidence does not support a direct mortality estimate: the included cohorts were neither powered for nor reported prostate-cancer-specific survival as a function of biopsy-to-RP upgrading, and the longest available surveillance series (Johns Hopkins, *n* = 1298) recorded grade reclassification but no prostate-cancer deaths over its follow-up [57]. What can be said is indirect but consistent. The grade ultimately present at prostatectomy—not the original biopsy grade—is the established driver of biochemical recurrence and cancer-specific outcome; a man whose true (RP) grade is GG ≥ 2 or GG ≥ 3 but who is assigned to surveillance on a falsely reassuring GG1 biopsy is therefore, in expectation, managed as lower risk than his biology warrants. Consistent with this, undetected cribriform/IDC—the morphology most often missed at biopsy—was independently associated with biochemical recurrence after RP (adjusted HR 2.14 [68]). The clinical inference is thus one of under-treatment from misclassification rather than a quantified excess mortality, and it reinforces the case for confirmatory imaging, targeted-plus-systematic re-sampling, and expert pathology review before a man is committed to surveillance. A definitive answer requires long-term prostate-cancer-specific mortality data stratified by biopsy-to-RP migration, identified here as a research priority (Section 4.5).

#### 4.3.2. Treatment Selection

The Weinstein net-benefit finding—that combined biopsy may carry net harm if downgrading is weighted more heavily than upgrading—has direct implications for the framing of treatment-selection conversations. Combined biopsy halves the odds of upgrading at RP versus systematic alone (OR 0.50) but doubles the odds of downgrading (OR 1.96) [22]; whether this is a net gain depends on whether the harm being optimized against is undertreatment (where upgrading matters most) or overtreatment (where downgrading matters most). PSMA-PET SUV_max_ demonstrates a robust monotonic relationship with RP grade [28], but the original meta-analysts explicitly cautioned against the use of single SUV_max_ thresholds for clinical decision-making in light of substantial between-study heterogeneity (I^2^ > 50% across all subgroups). Decisions about nerve-sparing, the extent of pelvic lymph-node dissection, eligibility for focal therapy, and the indication for adjuvant therapy all sit downstream of the index biopsy GG and inherit its uncertainty; this argues for shared decision-making that explicitly communicates the residual probability of grade migration rather than treating biopsy GG as a single point estimate.

#### 4.3.3. Biopsy Strategy

The hierarchy that emerges is consistent across study designs: in the post-mpMRI era, transperineal sampling improves concordance over transrectal sampling (Hagens: OR for concordance 1.33 [52]; Zattoni: multinational [53]; Kania: concordance 63% versus 49%, upgrading 16% versus 35% [55]); combined targeted-plus-systematic biopsy reduces upgrading further than targeted alone but at the cost of more frequent downgrading [22]; in-bore MRI-guided biopsy reaches intermediate concordance (64.2% in the largest single-center series [36]) and may be augmented by machine-learning prediction of upgrading. The targeted-biopsy overestimation finding in smaller tumors [21] is a brake on the uncritical adoption of “targeted only” pathways: even where mpMRI fusion is used, complementary systematic sampling preserves the sensitivity of the diagnostic pathway and, depending on patient-preference weights, may improve net benefit.

Three points deserve explicit clinical framing here. First, the inverse association between prostate volume and upgrading (pooled SMD −0.19 [37]) is best understood as a sampling-density and PSAD phenomenon rather than a protective effect of small glands: for a given PSA, a smaller prostate implies a higher PSAD, and a fixed number of biopsy cores samples a larger fraction of a small gland yet still readily misses a focal high-grade lesion when the cancer occupies a small absolute volume. The practical message for the clinician reading a biopsy and a scan together is that a small prostate with an apparently low-grade biopsy but a high PSAD and/or a PI-RADS 4–5 lesion should be treated as higher risk for occult upgrading rather than reassured by the gland size; prostate volume should be interpreted through PSAD rather than in isolation. Second, discordance is asymmetric across the grade spectrum: lower-grade biopsies are the ones most likely to be non-concordant by upgrading—biopsy GG1 carries the highest absolute upgrading risk (55–67% in dedicated cohorts [40,41]), because it has the most “room” to rise and is most vulnerable to under-sampling of a co-existing higher-grade focus, whereas the highest grades (GG5) can, by definition, only be concordant or downgraded, so their discordance is dominated by downgrading. Intermediate GG2–GG3 disease shows the widest between-cohort variability. The corollary is that the GG1/GG2 active-surveillance-eligible population is precisely where additional predictors and resampling yield the most decision-relevant information. Third, the evidence taken together supports a concrete best-practice pathway to minimize non-concordance: pre-biopsy multiparametric MRI with PI-RADS reporting, a transperineal route, and combined MRI-targeted plus systematic sampling (with targeted-only reserved for situations where the downgrading penalty is explicitly acceptable), read against expert or centrally reviewed pathology with mandatory cribriform/IDC and percentage-pattern-4 reporting. PSMA-PET and the moderate-certainty clinical predictors (PSAD, PI-RADS) are then used to flag the patients in whom residual discordance risk remains high.

#### 4.3.4. Clinically Actionable Versus Investigational Predictors

**Figure 6:**
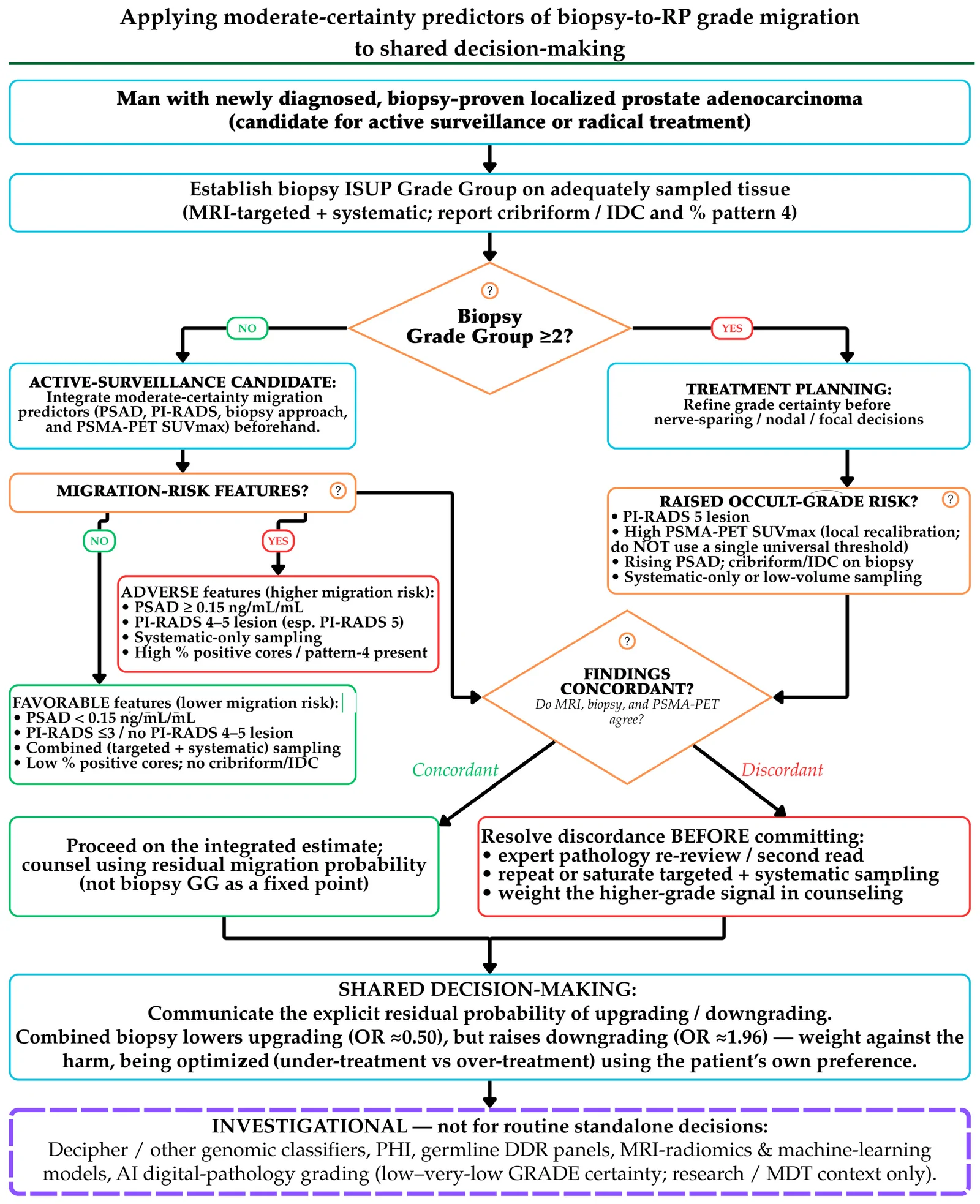
Clinical decision algorithm for applying the moderate-certainty predictors of biopsy-to-radical-prostatectomy ISUP Grade Group migration in shared decision-making. After the biopsy Grade Group is established on adequately sampled tissue, the pathway branches on whether it is ≥2: Grade Group 1 enters the active-surveillance limb (integrating PSA density, PI-RADS, biopsy approach, and PSMA-PET SUV_max_ to classify migration risk as favorable or adverse), while Grade Group ≥ 2 enters the treatment-planning limb (flagging a raised probability of occult higher-grade disease at RP). Both limbs converge on a concordance check among MRI, biopsy, and PSMA-PET: concordant findings support proceeding on the integrated estimate, whereas discordant findings prompt expert pathology re-review and repeated or saturated sampling before committing. The pathway terminates in shared decision-making, weighing the residual probability of upgrading versus downgrading (combined biopsy lowers upgrading, OR ≈ 0.50, but raises downgrading, OR ≈ 1.96) against the patient’s own preference. The lower banner indicates that molecular, genomic, and AI-based predictors remain of low-to-very-low GRADE certainty and investigational, suited to research or multidisciplinary-team contexts rather than routine standalone use. Abbreviations: GG = Grade Group; IDC = intraductal carcinoma; MDT = multidisciplinary team; OR = odds ratio; PI-RADS = Prostate Imaging–Reporting and Data System; PSA = prostate-specific antigen; PSAD = PSA density; PSMA-PET = prostate-specific membrane antigen positron emission tomography; RP = radical prostatectomy; SUV_max_ = maximum standardized uptake value.

To translate the certainty ratings into practice, the predictors can be arranged into a hierarchical taxonomy that maps onto four evidence tiers—validated clinical and imaging predictors, advanced imaging, molecular and genomic markers, and experimental artificial-intelligence tools—with descending readiness for routine use (Table 6). Only four predictors reached moderate certainty (PI-RADS category, PSAD, biopsy approach, and PSMA-PET SUV_max_); these can reasonably inform shared decision-making today, and the practical pathway for applying them—including when to intensify imaging or targeted biopsy, where PSAD and PI-RADS thresholds flag patients in whom an apparently surveillance-eligible biopsy may conceal higher-grade disease, and how to interpret a discordant MRI and biopsy—is set out as a decision aid, as specified in Figure 6. The remaining predictors are positioned as supportive (used within a multidisciplinary team rather than as standalone triggers) or as investigational and experimental, where the appropriate setting is prospective research with external validation rather than routine clinical decision-making. This actionable-versus-investigational distinction is intended to prevent the premature adoption of low- and very-low-certainty tools, in particular the machine-learning and radiomics models, which remain exploratory.

### 4.4. Strengths and Limitations

This review has several methodological strengths. It applied a pre-specified PICOTS structure adapted for prognostic-factor and prediction-model questions; it used three distinct risk-of-bias instruments matched to study design (QUIPS for prognostic-factor studies, PROBAST for prediction-model studies, and QUADAS-2 for diagnostic-accuracy studies); it adopted the GRADE framework adapted for prognostic research to rate certainty for each predictor–outcome pair; and it adhered to the SWiM reporting guidance [46] for narrative synthesis. The scope spans clinical, imaging, molecular/genomic, and AI-based predictors under a single contemporary date range (2010–2026) that captures the post-2014 ISUP, post-mpMRI, and post-PSMA-PET evidence base.

The limitations of this review must, however, be stated plainly. First, only one bibliographic database (MEDLINE via PubMed) was searched, supplemented by citation searching of recent relevant systematic reviews. Embase, the Cochrane Central Register of Controlled Trials, Scopus, and Web of Science were not searched. Single-database searching is known to reduce sensitivity, particularly for European urology and pathology journals indexed in Embase as well as for grey literature, conference proceedings, and registry data not consistently captured in MEDLINE; some primary studies and prediction-model reports relevant to biopsy-to-RP grade migration are likely to have been missed. This risk is not evenly distributed across predictor domains: radiomics, artificial-intelligence, and machine-learning studies are disproportionately reported in engineering, imaging-informatics, and conference-indexed venues that are captured more completely by Embase, IEEE Xplore, Scopus, and Web of Science than by MEDLINE (Section 2.3), and readers should bear in mind that the certainty ratings assigned to these emerging predictors in this review may partly reflect incomplete literature retrieval rather than a definitive appraisal of the underlying evidence base. We acknowledge that the prospectively registered protocol (PROSPERO CRD420261378765) outlined an intent to search PubMed/MEDLINE only; while this matches the search executed, it is below the multi-database threshold recommended by PRISMA 2020 Item 7 and the Cochrane Handbook §4.3.2 for clinical questions of this scope. A multi-database successor review is warranted (Section 4.5).

Second, the included primary studies were heterogeneous in their definitions of upgrading (any GG increase versus increase to ≥GG2 versus increase to ≥GG3, and modified 2005 Gleason versus 2014/2019 ISUP frameworks), in biopsy approach, in pathology re-review practices, in the version of PI-RADS used, and in the choice of multivariable model covariates. This clinical and methodological heterogeneity, together with the absence of patient-level data, was the principal reason no quantitative meta-analysis was attempted in this review; effect estimates were synthesized narratively and, where appropriate, alongside pre-existing pooled estimates from contemporary published meta-analyses. Third, PROBAST flagged the analysis domain as being at high risk of bias in five of eight included prediction-model studies, primarily because of insufficient sample size for the number of candidate predictors, the inadequate handling of missing data, and the absence of external validation. Seven of the eight models reported only internal validation; only the Cano Garcia SEER-based downgrading nomogram underwent independent external validation in an unaffiliated cohort [65]. Apparent discrimination metrics in the upper range—for example, the U.P.G.R.A.D.E. score AUC of 0.952 (95% CI 0.926–0.978) [64] and the Zhang radiomics AUC of 0.910 in training and validation [60]—are very likely optimistic estimates of out-of-sample performance, and none is currently ready for unmodified clinical deployment.

### 4.5. Future Directions

Four priorities follow from these findings. First, future modelling work should be explicitly multimodal rather than single-domain: the most promising contemporary studies in this review combined PSA and PSAD with PI-RADS, mpMRI radiomics, and either PSMA-PET parameters [63] or genomic-classifier scores [29], and the incremental value of any single emerging predictor over a well-specified clinical baseline was modest in studies that reported the comparison. The clinical question is not whether mpMRI radiomics, PSMA-PET, and AI-based digital pathology each add information—there is now reasonable evidence that each does—but how they should be integrated into a single calibrated risk estimate that supports shared decision-making.

Second, the prospective external-validation gap is the single largest barrier to the clinical translation of currently published prediction models. Of the eight multivariable models in our synthesis, only one was externally validated in an unaffiliated cohort [65]; the AI-based and radiomics models were uniformly internally validated only and reported incompletely against the TRIPOD+AI statement [72]. Adequately powered prospective external validation of the leading candidate models (combining contemporary mpMRI radiomics, PSMA-PET parameters where available, the Decipher classifier, and AI-based digital-pathology grading) is a near-term priority.

Third, geographic representation in the current evidence base is uneven. Of the 37 included primary studies, 11 enrolled patients in North America, 19 in Europe, five in East Asia, one in Australia, and one in another region; no included study was conducted in Sub-Saharan Africa or in Latin America at scale, and African-American men—for whom Vora and colleagues reported distinctive upgrading patterns [73]—remain under-represented in contemporary cohorts. The generalizability of currently reported predictor effect estimates to under-represented populations cannot be assumed. Two related points warrant explicit comment. On the question of whether race itself influences concordance: African-American men have a higher reported burden of anterior and apical tumors that are systematically harder to sample, and Vora et al. documented distinctive upgrading patterns in a large African-American population [73], so there is a plausible mechanism by which ancestry could be associated with greater biopsy-to-RP discordance—but this is most likely mediated by tumor location, access to and quality of mpMRI, and sampling technique rather than by ancestry per se, and the present evidence base is too sparse and too confounded to support race as an independent predictor of concordance. The responsible interpretation is that men from under-represented groups may be at higher risk of under-sampling and therefore of upgrading, which argues for equity of access to high-quality mpMRI and transperineal combined biopsy rather than for race-specific grade thresholds. On external validity more broadly, most prediction-model and biomarker studies originated in high-volume tertiary referral centers with subspecialty uropathology, expert mpMRI reading, established transperineal-fusion programs, and ready access to genomic platforms. Predictor effect sizes and, especially, model calibration derived under those conditions cannot be assumed to translate to lower-volume centers or to non-Western health systems with different imaging expertise, pathology-review practice, biopsy technique, and assay availability; this is a further reason the AI/radiomics and genomic predictors are not yet ready for routine, unmodified deployment and require local recalibration and prospective validation before adoption.

Fourth, the methodological constraints of the present review (single-database PubMed search, narrative synthesis only) themselves identify the case for an adequately resourced successor review. A multi-database (MEDLINE, Embase, CENTRAL, Scopus, Web of Science) review with formal quantitative pooling on the subset of homogeneous predictor–outcome pairs (PSAD, PI-RADS category, biopsy approach) where pooling is methodologically defensible would meaningfully strengthen the certainty ratings that the present synthesis supports. Such a review, ideally co-registered with prospective external validation studies of the leading prediction models, is the natural next step in this evidence stream.

Fifth, a question the included evidence could not answer—whether men assigned to active surveillance who are subsequently upgraded at prostatectomy carry a higher prostate-cancer-specific mortality—should be addressed directly: long-term, adequately powered cohorts reporting cancer-specific survival stratified by biopsy-to-RP grade migration (rather than by biopsy grade alone) are needed to quantify the clinical cost of misclassification and would convert the present mechanistic inference into an actionable risk estimate for surveillance counselling.

## 5. Conclusions

In contemporary cohorts assembled in the post-mpMRI, post-2014-ISUP era, biopsy-to-radical-prostatectomy ISUP GG discordance remains substantial: concordance is approximately 58–63% in the largest registries, upgrading affects 25–32% of patients overall and up to 55–67% of biopsy GG1 patients in dedicated cohorts, and downgrading has increased to approximately 15% with widespread adoption of MRI-targeted biopsy. The 2014 ISUP five-tier GG system has reduced the apparent rate of clinically significant upgrading without a corresponding increase in downgrading.

Of the predictors examined in this review, four reached moderate GRADE certainty for biopsy-to-RP grade migration: PI-RADS category, biopsy approach (combined versus systematic), PSMA-PET maximum standardized uptake value, and PSAD. Cribriform and IDC at biopsy, the Decipher genomic classifier, the PHI, and MLMs reached low certainty. Germline mutations as direct predictors of biopsy-to-RP upgrading reached very low certainty. No predictor reached high certainty.

Methodological readiness for clinical translation is uneven. Only one of the eight included multivariable prediction models has been externally validated in an unaffiliated cohort, and PROBAST flagged the analysis domain as high risk of bias in five of the eight. The contemporary evidence supports incorporating PI-RADS category, biopsy approach, and PSAD into shared decision-making about active surveillance, treatment selection, and biopsy strategy; by contrast, emerging molecular and, in particular, AI- and radiomics-based predictors remain exploratory. On current evidence, they are not ready for routine, standalone clinical decision-making and should be confined to research and multidisciplinary-team settings; they require prospective, multicenter external validation with full calibration reporting in adequately powered, geographically representative cohorts—and, for tools developed in specialized centers, local recalibration—before any routine clinical adoption. The single-database scope of the present review further indicates the need for a multi-database successor synthesis with formal quantitative pooling on the homogeneous predictor–outcome pairs for this clinically important question.

## Figures and Tables

**Table 1:** Selected characteristics and primary outcome rates of included studies, with supplementary input (representative subset [14,15,18,21,26,36,40,41,42,49,50,51,52,53,54,55,56]; all primary studies presented in the main text by domain).

Study (Year)	Country/Setting	*n* Paired Bx–RP	Biopsy Approach	Concordance (%)	Upgrading (%)	Downgrading (%)	Ref.
Epstein (2012)	USA, Hopkins	7643	Systematic	—	36.3 *	—	[14]
De Nunzio (2018)	Italy/Germany 4-center	9703	Systematic	κ = 0.36	19.5 †	7.7	[42]
Bullock (2019)	UK BAUS Registry	17,598	Systematic	58.9	25.5	15.6	[15]
Ahdoot (2020)	USA NCI Trio study	404	Combined Bx	—	14.4	NS	[26]
Ahdoot (2020)	USA NCI Trio study	404	MRI-targeted	—	30.9	NS	[26]
Ahdoot (2020)	USA NCI Trio study	404	Systematic	—	41.6	NS	[26]
Soenens (2020)	Belgium Be-RALP	8021	Systematic (RARP)	62.9	27.3	—	[49]
Su (2020)	USA, Hopkins	1325 LR	Systematic	—	55.1 ‡	—	[40]
Wang X (2021)	China nomogram	198	Systematic 24-core	44.4	41.4	—	[50]
van der Slot (2022)	Netherlands GG2	196	mpMRI + TBx + SBx	—	54.1 ¶	—	[51]
Hagens (2024)	Netherlands TR vs. TP	1058	TR vs. TP post-MRI	—	—	—	[52]
Zattoni (2023)	EAU-YAU multinational	1282	TR-TBx vs. TP-TBx	—	—	—	[53]
Kesch (2023)	EU multicenter	475	TBx + SBx	—	39.0	—	[18]
Liss (2024)	USA prospective GG1	285	Mixed	—	67.0 ‡	—	[41]
Ozbozduman (2024)	Turkey in-bore MRGB	95	In-bore MRI biopsy	64.2	28.4	7.4	[36]
Kroon (2025)	EU Anser collab.	616	Targeted	58	27	15	[21]
Baboudjian (2025)	EU multicenter AS-GG2	139 (28 RPs)	TR-TBx vs. TP-TBx	—	46 §	—	[54]
Kania (2025)	Poland ComBx	68 ComBx/ 89 TRUS-Bx	ComBx vs. TRUS-Bx	63/49	16/35	—	[55]
Jain (2025)	Australia GG2	243	TP post-MRI	—	16.9	—	[56]

* Upgrading of biopsy Gleason score 5–6 to RP Gleason score ≥ 7. † Clinically significant upgrading using the five-tier 2014 ISUP Grade Group system (downstaging from 24.0% with the 2005 modified Gleason scoring system). ‡ Upgrading of biopsy GG1 to RP GG ≥ 2. § Of 139 GG2 patients managed with active surveillance after pre-biopsy mpMRI, 28 underwent RP and 13 (46%) had adverse features (GG ≥ 3 and/or pT ≥ 3a); very aggressive disease (GG ≥ 4 and/or pT ≥ 3b and/or pN 1) was present in two (7%). Included as supplementary references—cohort under the pre-specified ≥ 50 paired-case threshold. ¶ Composite endpoint of GG ≥ 2+ at RP (GG2 with cribriform/intraductal carcinoma at RP, or any GG > 2 at RP) per van der Slot et al. (2022) [51]. Abbreviations: BAUS = British Association of Urological Surgeons; Be-RALP = Belgian Robot-Assisted Laparoscopic Prostatectomy registry; Bx = biopsy; ComBx = combined (MRI-targeted plus systematic) transperineal fusion biopsy; EAU-YAU = European Association of Urology—Young Academic Urologists; GG = (ISUP) Grade Group; LR = low risk; MRGB = MRI-guided biopsy; NS = not significant; RP = radical prostatectomy; SBx = systematic TRUS-guided biopsy; TBx = MRI-targeted biopsy; TP = transperineal; TR(US) = transrectal (ultrasound); TRUS-Bx = TRUS-guided biopsy.

**Table 2:** Structured subgroup and sensitivity synthesis of factors modifying biopsy-to-radical-prostatectomy ISUP Grade Group migration.

Subgroup Axis	Comparison	Direction of Effect on Biopsy-to-RP Migration	Certainty/Notes
Biopsy sampling	MRI-targeted or combined vs. systematic	Targeted and combined sampling lower upgrading (combined OR < 1 [22]); combined sampling roughly doubles downgrading	Moderate
Imaging era	Pre-MRI (systematic-only) vs. contemporary mpMRI-directed era	Upgrading rates fall but persist in the MRI era; downgrading more frequent where combined sampling is used	Moderate
Grading-system era	2005 modified Gleason (7) vs. 2014 ISUP GG (27) vs. 2019 ISUP (3)	Raw migration rates not directly comparable; 2019 cribriform/IDC reporting refines but does not remove discordance	Low (indirectness)
Baseline risk mix	AS-eligible/Grade Group 1 vs. mixed-risk cohorts	Upgrading concentrated in GG1 (55–67% [40,41]); mixed cohorts dilute apparent population rates	Moderate
Center structure	Single-center derivation vs. multicenter/externally validated	Single-center estimates systematically optimistic; multicenter/external estimates more conservative	Low–moderate

Abbreviations: AS = active surveillance; combined = combined MRI-targeted plus systematic biopsy; GG = (ISUP) Grade Group; GG1 = Grade Group 1; IDC = intraductal carcinoma; ISUP = International Society of Urological Pathology; mpMRI = multiparametric magnetic resonance imaging; MRI = magnetic resonance imaging; OR = odds ratio; RP = radical prostatectomy. Numbers in parentheses in the “Comparison” column (7, 27, 3) indicate the number of included primary studies using each grading framework. “Certainty” reflects the GRADE rating for the consistency and directness of the subgroup signal, not a formal pooled estimate; subgroups were pre-specified to organize narrative synthesis rather than as formal statistical comparisons (Section 2.6). Directions are qualitative summaries of consistently reported signals; no quantitative pooling was performed in this review.

**Table 3:** Multivariable prediction models for biopsy-to-radical-prostatectomy ISUP Grade Group migration.

First Author (Year)	*n*	Model/Predictors	AUC/C-Index	External Validation	Ref.
Wang X (2021)	198	Chinese nomogram: PSA, GPC, cT, PI-RADS	0.735 (apparent); 0.726 (bias-corr.)	No	[50]
Karamık (2024)	235	U.P.G.R.A.D.E. score: prior biopsy, PSA, GPC, PI-RADS, age, biopsy–RP delay, % positive cores	0.952 (95% CI 0.926–0.978) ***	No	[64]
Zhang (2020)	166	MRI radiomics + clinical: T2W + ADC + DCE radiomics, cT, biopsy–RP delay	0.910 (training + validation)	No	[60]
Marvaso (2024)	949	Gradient-boosted ML: Clinical + Radiological + Radiomic	0.73–0.96 across 6 endpoints	No	[61]
Ozbozduman (2024)	95	Pre-biopsy + MRGB clinical features	0.856	No	[36]
Soeterik (2024)	605	PSMA-PET parameters (multicentric): PSMA-volume, SUV_max_, PSMA-total, PSA, cT	Improves over base clinical	No *	[63]
Cano Garcia (2023)	Large (SEER)	SEER downgrading nomogram: Clinical + biopsy + treatment-era covariates	External-validation discrimination	Yes **	[65]
Wang G (2023)	356	Bayesian network: age, tPSA, PSAD, prostate volume, % positive cores, PI-RADS, cT	—	No	[62]

* Multinational seven-center development cohort (no separate external-validation cohort reported in the source publication). ** External validation performed in a single tertiary cohort. *** Apparent (development-cohort) AUC; not bias-corrected. Apparent AUCs in development cohorts of this size, particularly in the absence of external validation and full calibration reporting, are likely to overestimate out-of-sample discrimination (see Section 4.4). Abbreviations: ADC = apparent diffusion coefficient; AUC = area under the receiver operating characteristic curve; bias-corr. = bias-corrected (bootstrap-adjusted apparent estimate); CI = confidence interval; cT = clinical T stage; DCE = dynamic contrast-enhanced (MRI sequence); GPC = greatest percentage of cancer per core; ML = machine learning; MRGB = MRI-guided biopsy; MRI = magnetic resonance imaging; *n* = number of patients in the development cohort; PI-RADS = Prostate Imaging–Reporting and Data System; PSA = prostate-specific antigen; PSAD = prostate-specific antigen density; PSMA = prostate-specific membrane antigen; PSMA-PET = prostate-specific membrane antigen positron emission tomography; Ref. = reference number in the bibliography; RP = radical prostatectomy; SEER = Surveillance, Epidemiology, and End Results (USA registry); SUV_max_ = maximum standardized uptake value (on PSMA-PET); T2W = T2-weighted (MRI sequence); tPSA = total prostate-specific antigen; U.P.G.R.A.D.E. = name of a published prediction score (Karamık et al. 2024 [64]). Areas under the receiver operating characteristic curve (AUCs) are reported as published in the corresponding source publications; calibration metrics (Hosmer–Lemeshow, calibration-in-the-large, calibration slope) are also reported in the source publications and are not duplicated here.

**Table 4:** Summary of risk-of-bias judgements across included studies, by predictor domain.

Predictor Domain	Tool Used	No. of Studies	Low Risk	Moderate Risk	High Risk	Principal Concerns
Clinical variables (PSA, PSAD, age, %cores)	QUIPS	21	5	12	4	Confounding; biopsy approach reporting
mpMRI/PI-RADS	QUIPS, QUADAS-2	11	2	7	2	PI-RADS version heterogeneity; reader experience
MRI radiomics/ML	PROBAST	5 †	0	2	3	Sample size; missing external validation
PSMA-PET	QUIPS, QUADAS-2	4	1	2	1	Tracer heterogeneity; selection bias
Biopsy approach	QUIPS	9	3	5	1	Selection bias; era of mpMRI adoption
Cribriform/IDC	QUIPS	5	1	3	1	Reporting standardization; pre-2019 ISUP era
Genomic/serum/urinary biomarkers	QUIPS, PROBAST	4	1	2	1	Small samples; commercial assay heterogeneity
Germline mutations	QUIPS	1	1	0	0	Single prospective study
All prediction models (composite)	PROBAST	8	1	2	5	Analysis domain (sample size, missing data, no external validation)

Abbreviations: IDC = intraductal carcinoma; ISUP = International Society of Urological Pathology; ML = machine learning; mpMRI = multiparametric magnetic resonance imaging; PI-RADS = Prostate Imaging–Reporting and Data System; PROBAST = Prediction model Risk Of Bias ASsessment Tool; PSA = prostate-specific antigen; PSAD = prostate-specific antigen density; PSMA-PET = prostate-specific membrane antigen positron emission tomography; QUADAS-2 = Quality Assessment of Diagnostic Accuracy Studies, version 2; QUIPS = Quality In Prognosis Studies tool. Categorical counts reflect the number of contributing studies in each domain; some studies appear in more than one row because they evaluated more than one predictor or used more than one risk-of-bias instrument. † Subset of the 8 prediction-model studies in Table 3; the remaining 3 studies (Wang X et al. 2021 [50]; Karamık et al. 2024 [64]; Cano Garcia et al. 2023 [65]) are clinical nomograms assessed separately.

**Table 5:** GRADE summary of findings for emerging predictors of biopsy-to-radical-prostatectomy ISUP Grade Group migration.

Predictor	Outcome	GRADE Certainty	Key Reasons for Rating	No. of Studies	Effect Direction and Consistency	Overall Risk of Bias
PI-RADS category	UpG at RP	Moderate ⊕⊕⊕○	Consistent direction across studies; Downgraded once for residual confounding	Multiple RCTs + 2 meta-analyses [22,27]	Consistent: higher PI-RADS → higher UpG; mpMRI pathway reduces (not eliminates) UpG	Low–moderate
Biopsy approach (combined vs. systematic)	UpG at RP	Moderate ⊕⊕⊕○	Pooled estimates from two contemporary meta-analyses; Downgraded for indirectness (downgrading penalty)	2 meta-analyses (up to 26 studies/6638 patients) [22]	Consistent: combined biopsy lowers UpG (OR < 1) but raises downgrading	Low–moderate
PSMA-PET SUV_max_	UpG at RP	Moderate ⊕⊕⊕○	Robust monotonic relationship; Downgraded once for inter-study heterogeneity (I^2^ > 50%)	1 meta-analysis (23 studies) [28]	Consistent monotonic rise of SUV_max_ with RP grade	Moderate (I^2^ > 50%)
PSA density	UpG at RP	Moderate ⊕⊕⊕○	Largest SMD in pooled analysis [37]; Consistent in contemporary multivariable studies	1 pooled meta-analysis [37] + multivariable cohorts	Consistent: largest pooled SMD among continuous predictors; same direction across studies	Low–moderate
Cribriform/IDC at biopsy	UpG at RP	Low ⊕⊕○○	Downgraded for risk of bias and indirectness related to inconsistent reporting standards	Few studies [51,66,67,68]; inconsistent reporting standards	Direction consistent (presence → UpG); magnitude varies widely	Moderate–high
Decipher genomic classifier	UpG at RP	Low ⊕⊕○○	Few primary studies; Small samples; Commercial-assay heterogeneity	Few primary studies [29,31]; small samples	Positive but imprecise independent association (e.g., OR > 1, wide CI)	Moderate–high
Prostate Health Index (PHI/PHID)	UpG at RP	Low ⊕⊕○○	Three primary studies; Heterogeneous cut-offs	3 primary studies [30,69,70]	Direction consistent; cut-offs heterogeneous	Moderate
Machine-learning/radiomics models	UpG at RP	Very Low ⊕○○○	Internal validation only; 3 of 5 high analysis-domain risk of bias (PROBAST); Serious imprecision + indirectness (single-center Chinese/Turkish/Italian cohorts); Incomplete TRIPOD+AI reporting	5 studies [36,60,61,62,63]	Apparent reduction in migration in-sample; not reproduced externally	High (PROBAST analysis domain)
AI digital-pathology grading	UpG at RP	Low ⊕⊕○○	Pathologist-level reading demonstrated; Downstream effect on biopsy–RP migration not yet quantified prospectively	2 included + 3 supporting reports [33,34,35,36]	Pathologist-level grading shown; biopsy–RP migration effect not yet quantified prospectively	Moderate–high
Germline DNA-damage-repair mutations	UpG at RP	Very low ⊕○○○	Single large prospective study negative [41]; Imprecision and indirectness	1 large prospective study (negative) [41]	No independent association with biopsy-to-RP UpG	Moderate (indirectness, imprecision)
Clinical nomograms (multivariable)	UpG at RP	Low ⊕⊕○○	1 of 3 externally validated (AUC 0.72 [65]); 2 of 3 high PROBAST analysis risk of bias; Single-center derivation cohorts; Indirectness	3 studies [50,64,65]	Moderate discrimination (AUC ~0.72); variable across cohorts	Moderate–high

Filled circles (⊕) denote Grading of Recommendations, Assessment, Development and Evaluations (GRADE) certainty ratings: ⊕⊕⊕○ = moderate certainty; ⊕⊕○○ = low certainty; ⊕○○○ = very low certainty. Domains assessed in deriving these ratings: risk of bias, inconsistency (between-study heterogeneity), indirectness (of population, predictor, outcome, or comparator), imprecision (of pooled estimates), publication bias, and phase of investigation (model development versus internal validation versus external validation). Abbreviations: AI = artificial intelligence; GRADE = Grading of Recommendations, Assessment, Development and Evaluations; IDC = intraductal carcinoma; mpMRI = multiparametric magnetic resonance imaging; PHI = Prostate Health Index; PHID = Prostate Health Index density; PI-RADS = Prostate Imaging–Reporting and Data System; PSA = prostate-specific antigen; PSMA-PET = prostate-specific membrane antigen positron emission tomography; RP = radical prostatectomy; SMD = standardized mean difference; SUV_max_ = maximum standardized uptake value; TRIPOD+AI = Transparent Reporting of a multivariable prediction model for Individual Prognosis Or Diagnosis—Artificial Intelligence extension; UpG = Upgrading.

**Table 6:** Hierarchical taxonomy of predictors of biopsy-to-radical-prostatectomy ISUP Grade Group migration, ordered by readiness for clinical use.

Tier	Predictors	Current Role	Certainty
1. Validated clinical and imaging (actionable now)	PI-RADS category; PSA density; biopsy approach (combined/targeted); PSMA-PET SUV_max_	Incorporate into shared decision-making and active-surveillance eligibility; intensify imaging/targeted biopsy in flagged patients	Moderate
2. Advanced/supportive (validation stage)	Cribriform and IDC morphology at biopsy; multivariable clinical nomograms; AI digital-pathology grading	Use as supportive flags within a MDT; not standalone decision triggers	Low
3. Molecular/genomic (investigational)	Decipher and other tissue genomic classifiers; PHI and serum/urinary biomarkers	Selected cases or research; not for routine grade-migration decisions	Low
4. Experimental AI (not for routine use)	MRI radiomics and machine-learning models; germline DNA-damage-repair mutations as direct predictors	Prospective research with external validation only	Very low

Abbreviations: AI = artificial intelligence; IDC = intraductal carcinoma; MDT = multidisciplinary team; MRI = magnetic resonance imaging; PHI = Prostate Health Index; PI-RADS = Prostate Imaging–Reporting and Data System; PSA = prostate-specific antigen; PSMA-PET = prostate-specific membrane antigen positron emission tomography; SUV_max_ = maximum standardized uptake value. Certainty reflects the GRADE ratings summarized in Table 5; the clinical pathway for the moderate-certainty (Tier 1) predictors is shown in Figure 6. Tiers are ordered by readiness for routine clinical use, from validated and actionable (Tier 1) to experimental (Tier 4); tier assignment reflects current evidence maturity and is not a permanent classification.

## Data Availability

Data available on request.

## Supplementary Materials

The following supporting information can be downloaded at: https://www.mdpi.com/article/10.3390/biomedicines14081736/s1, Supplementary File S1: PubMed/MEDLINE Search Strategy; Supplementary File S2: QUIPS risk-of-bias appraisal of prognostic-factor cohort studies; Supplementary File S3: PROBAST risk-of-bias and applicability appraisal of prediction-model studies; Supplementary File S4: QUADAS-2 risk-of-bias and applicability appraisal of diagnostic-accuracy and biopsy-vs-RP concordance studies; Supplementary File S5: PRISMA 2020 checklist.

## Abbreviations

The following abbreviations are used in this manuscript:

4Kscore	four-kallikrein panel blood test (proprietary name)
ADC	apparent diffusion coefficient
AI	artificial intelligence
AUA	American Urological Association
AUC	area under the receiver operating characteristic curve
BAUS	British Association of Urological Surgeons
Be-RALP	Belgian Robot-Assisted Laparoscopic Prostatectomy registry
BMI	body mass index
Bx	biopsy
CAPRA	Cancer of the Prostate Risk Assessment (score)
CENTRAL	Cochrane Central Register of Controlled Trials
CHARMS	Critical Appraisal and Data Extraction for Systematic Reviews of Prediction Modelling Studies
CI	confidence interval
C-index	concordance index
ComBx	combined (MRI-targeted plus systematic) biopsy
cT	clinical T stage
CT	computed tomography
DNA	deoxyribonucleic acid
EAU	European Association of Urology
EAU-YAU	EAU Young Academic Urologists
EPE	extraprostatic extension
ExoDx EPI	ExoDx Prostate IntelliScore (urinary exosome assay)
FDA	(US) Food and Drug Administration
GG	(ISUP) Grade Group
GPS	Genomic Prostate Score (Oncotype DX)
GRADE	Grading of Recommendations, Assessment, Development and Evaluations
GS	Gleason score
HR	hazard ratio
IDC	intraductal carcinoma
IQR	interquartile range
ISUP	International Society of Urological Pathology
MLM	machine-learning model
mpMRI	multiparametric magnetic resonance imaging
MRI	magnetic resonance imaging
MRI-FIRST	MRI-FIRST randomized trial
MUSIC	Michigan Urological Surgery Improvement Collaborative
NCCN	National Comprehensive Cancer Network
NCI	(USA) National Cancer Institute
NLR	neutrophil-to-lymphocyte ratio
NRG	NRG Oncology (cooperative trials group)
OR	odds ratio
PANDA	Prostate cANcer graDe Assessment (challenge)
PCA3	prostate cancer antigen 3
PHI(D)	Prostate Health Index (Density)
PI-RADS	Prostate Imaging–Reporting and Data System
PICOTS	Population, Index prognostic factor, Comparator, Outcome, Timing, Setting (review framework)
PNI	perineural invasion
PRIMARY	PRIMARY score (intraprostatic PSMA-PET assessment)
PRISMA	Preferred Reporting Items for Systematic Reviews and Meta-Analyses
PRISMA-P	PRISMA for Protocols
PROBAST	Prediction model Risk Of Bias Assessments Tool
PROMIS	PROstate MR Imaging Study trial
PROSPERO	International Prospective Register of Systematic Reviews
PSA/PSAD	prostate-specific antigen/PSA density
PSMA	prostate-specific membrane antigen
PSMA-PET	PSMA positron emission tomography
pT	pathological T stage
QUADAS-2	Quality Assessment of Diagnostic Accuracy Studies, version 2
QUIPS	Quality In Prognosis Studies (tool)
RARP	robot-assisted radical prostatectomy
RP	radical prostatectomy
RTOG	Radiation Therapy Oncology Group
SEER	Surveillance, Epidemiology, and End Results (program)
SelectMDx	urinary biomarker assay (proprietary name)
SMD	standardized mean difference
SUV_max_	maximum standardized uptake value
SWiM	Synthesis Without Meta-analysis (reporting guideline)
TRIPOD	Transparent Reporting of a multivariable prediction model for Individual Prognosis or Diagnosis
TRIPOD+AI	TRIPOD+Artificial Intelligence extension
TRUS	transrectal ultrasound
TRUS-Bx	transrectal ultrasound-guided biopsy
UK	United Kingdom
USA	United States of America
