# Supplementary material for: ISUP Grade Group Migration Between Prostate Biopsy and Radical Prostatectomy: A Systematic Review of Emerging Predictors
Source: Biomedicines. 2026 Jul 31;14(8):1736. doi: 10.3390/biomedicines14081736 (PMC13509513; doi:10.3390/biomedicines14081736)
Supplement: Supplementary file 1 [file biomedicines-14-01736-s001.zip › PRISMA_2020_checklist_filled.pdf]

PRISMA 2020 Checklist

Section and Topic	Item #	Checklist item	Location where item is reported
**TITLE**			
Title	1	Identify the report as a systematic review.	Title page; the designation "Systematic Review" appears immediately above the title.
**ABSTRACT**			
Abstract	2	See the PRISMA 2020 for Abstracts checklist.	Abstract (structured: Background and Objectives; Materials and Methods; Results; Conclusions).
**INTRODUCTION**			
Rationale	3	Describe the rationale for the review in the context of existing knowledge.	Section 1 (Introduction), paragraphs 1–4.
Objectives	4	Provide an explicit statement of the objective(s) or question(s) the review addresses.	Section 1 (Introduction), final paragraph (five numbered aims i–v); PICOTS framing in Section 2.2.
**METHODS**			
Eligibility criteria	5	Specify the inclusion and exclusion criteria for the review and how studies were grouped for the syntheses.	Section 2.2 (Eligibility Criteria and Review Question).
Information sources	6	Specify all databases, registers, websites, organisations, reference lists and other sources searched or consulted to identify studies. Specify the date when each source was last searched or consulted.	Section 2.3 (Information Sources and Search Strategy). MEDLINE via PubMed only, supplemented by citation searching of recent systematic reviews and meta-analyses; date range 1 January 2010 to the date of the final search update before manuscript preparation. Single-database scope acknowledged as a limitation in Section 4.4.
Search strategy	7	Present the full search strategies for all databases, registers and websites, including any filters and limits used.	Section 2.3, paragraph 2 (three-block strategy: population × grading/migration × intervention/predictor; MeSH and free-text terms; PubMed-native truncation and field tags; English-language restriction). The full PubMed query string, conceptual-block structure, hit counts by block, and citation-searching procedure are provided in Supplementary File S1 (Search Strategy).
Selection process	8	Specify the methods used to decide whether a study met the inclusion criteria of the review, including how many reviewers screened each record and each report retrieved, whether they worked independently, and if applicable, details of automation tools used in the process.	Section 2.4 (Study Selection and Data Extraction), paragraph 1. Title-and-abstract and full-text screening performed independently and in duplicate by two reviewers, blinded to each other’s decisions; inter-rater agreement statistics were not formally computed; disagreements resolved by discussion, with provision for adjudication by a third reviewer. EndNote 21 used for deduplication; Rayyan used for screening. No automation/ML triage tools used.
Data collection process	9	Specify the methods used to collect data from reports, including how many reviewers collected data from each report, whether they worked independently, any processes for obtaining or confirming data from study investigators, and if applicable, details of automation tools used in the process.	Section 2.4, paragraphs 2–3. Data extraction performed independently and in duplicate by two reviewers using a pre-piloted standardized Excel form; discrepancies resolved by discussion against the source publication, with third-reviewer adjudication available. Corresponding authors were not contacted for unpublished data; only published full-text and supplementary materials extracted.
Data items	10a	List and define all outcomes for which data were sought. Specify whether all results that were compatible with each outcome domain in each study were sought (e.g. for all measures, time points, analyses), and if not, the methods used to decide which results to	Section 2.2, paragraph 2 (outcomes). Primary outcome: ISUP Grade Group migration (upgrading/downgrading) between biopsy and radical prostatectomy. Secondary outcomes: clinically

Section and Topic	Item #	Checklist item	Location where item is reported
		collect.	significant upgrading (defined a priori), concordance, predictor effect estimates, prediction-model performance metrics. Adjusted estimates prioritised when both adjusted and unadjusted were reported.
	10b	List and define all other variables for which data were sought (e.g. participant and intervention characteristics, funding sources). Describe any assumptions made about any missing or unclear information.	Section 2.4, paragraph 2 (data items list). Comprehensive list of study, population, biopsy, imaging, molecular, RP, outcome, and methodological characteristics. No imputation of missing data; only data reported in the published full text or its supplementary materials were extracted.
Study risk of bias assessment	11	Specify the methods used to assess risk of bias in the included studies, including details of the tool(s) used, how many reviewers assessed each study and whether they worked independently, and if applicable, details of automation tools used in the process.	Section 2.5 (Risk of Bias Assessment). QUIPS for prognostic-factor studies, PROBAST for prediction-model studies, QUADAS-2 for diagnostic-accuracy studies. Assessed independently and in duplicate by two reviewers; inter-rater agreement statistics were not formally computed; disagreements were resolved by discussion, with adjudication by a third reviewer available.
Effect measures	12	Specify for each outcome the effect measure(s) (e.g. risk ratio, mean difference) used in the synthesis or presentation of results.	Section 2.6 (Data Synthesis and Certainty of Evidence), paragraph 1. Prevalence proportions for concordance, upgrading, and downgrading; odds ratios with 95% CIs for binary predictor associations; AUC or C-index with 95% CIs for prediction-model discrimination. Hazard ratios extracted narratively but not pooled with odds ratios.
Synthesis methods	13a	Describe the processes used to decide which studies were eligible for each synthesis (e.g. tabulating the study intervention characteristics and comparing against the planned groups for each synthesis (item #5)).	Section 2.6, paragraph 2. Pre-specified narrative subgroups (biopsy approach; ISUP grading era; biopsy GG at diagnosis; central versus local pathology; geographic region) used to organise synthesis.
	13b	Describe any methods required to prepare the data for presentation or synthesis, such as handling of missing summary statistics, or data conversions.	Section 2.6, paragraph 1. Effect estimates extracted as reported. Adjusted estimates preferred over unadjusted when both were available. No data conversions or imputation performed.
	13c	Describe any methods used to tabulate or visually display results of individual studies and syntheses.	Section 2.7 (Software); Figures 2–4; Tables 1–3. Figures generated in Python 3.11 with matplotlib (forest-plot-style display of pooled estimates from prior meta-analyses; PSMA SUVmax distributions; clinical-predictor associations). Tabular summaries by predictor domain.
	13d	Describe any methods used to synthesize results and provide a rationale for the choice(s). If meta-analysis was performed, describe the model(s), method(s) to identify the presence and extent of statistical heterogeneity, and software package(s) used.	Section 2.6, paragraph 2. No quantitative meta-analyses were performed in this review; rationale: anticipated heterogeneity in biopsy technique, imaging protocol, pathology re-review, predictor definitions, and risk-group case-mix. Synthesis conducted narratively in accordance with the SWiM (Synthesis Without Meta-analysis) reporting guidance. Where contemporary published systematic reviews and meta-analyses had already pooled comparable studies for a given predictor, their pooled estimates are reproduced and clearly attributed rather than re-derived.
	13e	Describe any methods used to explore possible causes of heterogeneity among study results (e.g. subgroup analysis, meta-regression).	Section 2.6, paragraph 2. Pre-specified narrative subgroups (not formal statistical comparisons). No meta-regression performed (no

Section and Topic	Item #	Checklist item	Location where item is reported
			quantitative pooling).
	13f	Describe any sensitivity analyses conducted to assess robustness of the synthesized results.	Not applicable — no quantitative pooling, therefore no sensitivity analyses. Robustness was instead assessed qualitatively against per-predictor risk-of-bias judgements (Section 3.11) and GRADE certainty (Section 3.12).
Reporting bias assessment	14	Describe any methods used to assess risk of bias due to missing results in a synthesis (arising from reporting biases).	Section 2.6, paragraph 3. Formal statistical tests for publication or reporting bias (e.g., funnel plots, Egger's test, trim-and-fill) not applicable in the absence of quantitative pooling. Potential for selective reporting in primary studies and in cited prior meta-analyses acknowledged narratively.
Certainty assessment	15	Describe any methods used to assess certainty (or confidence) in the body of evidence for an outcome.	Section 2.6, paragraph 3. GRADE framework adapted for prognostic-factor research, applied independently by two reviewers across the domains of risk of bias, inconsistency, indirectness, imprecision, publication bias, and phase of investigation. Categories: high / moderate / low / very low. Disagreements resolved by discussion.
**RESULTS**			
Study selection	16a	Describe the results of the search and selection process, from the number of records identified in the search to the number of studies included in the review, ideally using a flow diagram.	Section 3.1 (Study Selection); Figure 1 (PRISMA 2020 flow diagram). Exact counts are reported at all flow-diagram stages (847 records identified via PubMed/MEDLINE, 38 additional records via citation searching of eight prior systematic reviews and meta-analyses, 23 duplicates removed, 862 screened, 691 excluded at title/abstract, 171 sought for retrieval, 12 not retrievable in English full text, 159 assessed for eligibility, 122 excluded with reasons, 37 primary studies included).
	16b	Cite studies that might appear to meet the inclusion criteria, but which were excluded, and explain why they were excluded.	Section 3.1; Figure 1 (excluded-with-reasons box). Reasons for full-text exclusion enumerated in Section 3.1 (no paired biopsy–RP comparison; <50 paired cases; active surveillance without subsequent RP; cohort overlap with a larger included report; pre-2005 Gleason data only; full text not available in English; etc.).
Study characteristics	17	Cite each included study and present its characteristics.	Section 3.2 (Characteristics of Included Studies); Table 1. Thirty-seven included primary studies plus eight contemporary published systematic reviews and meta-analyses [13,16,22,27,28,37,44,45] cited and characterised; representative subset displayed in Table 1.
Risk of bias in studies	18	Present assessments of risk of bias for each included study.	Section 3.11 (Risk of Bias in Included Studies); Table 3 (risk-of-bias judgements summarised by predictor domain).
Results of individual studies	19	For all outcomes, present, for each study: (a) summary statistics for each group (where appropriate) and (b) an effect estimate and its precision (e.g. confidence/credible interval), ideally using structured tables or plots.	Sections 3.3–3.10; Tables 1–2; Figures 2–4. Concordance, upgrading, and downgrading rates and per-predictor effect estimates with 95% CIs reported throughout. Pooled estimates from cited prior meta-analyses presented graphically in Figures 2–4.
Results of	20a	For each synthesis, briefly summarise the characteristics and risk of bias among	Sections 3.3–3.10; Section 3.11 (cross-cutting risk of bias); Table

Section and Topic	Item #	Checklist item	Location where item is reported
syntheses		contributing studies.	3. Each predictor-domain subsection (3.4 clinical; 3.5 imaging; 3.6 biopsy approach; 3.7 cribriform/IDC; 3.8 genomic/biomarker; 3.9 AI; 3.10 prediction models) summarises contributing studies and their methodological features, with risk of bias cross-referenced to Table 3.
	20b	Present results of all statistical syntheses conducted. If meta-analysis was done, present for each the summary estimate and its precision (e.g. confidence/credible interval) and measures of statistical heterogeneity. If comparing groups, describe the direction of the effect.	Not applicable — no new statistical synthesis was performed in this review. Pooled estimates from contemporary published meta-analyses are reproduced and clearly attributed: Section 3.4 (Wang Y 2023 [37]; Wang X 2021 [44]); Section 3.5 (Chen DC 2025 [28]); Section 3.6 (Goel 2020 [27]; Weinstein 2023 [22]); Section 3.7 (Diamand 2025 [16]).
	20c	Present results of all investigations of possible causes of heterogeneity among study results.	Not applicable — no quantitative heterogeneity assessment performed because no pooled meta-analysis was conducted. Sources of clinical and methodological heterogeneity are discussed narratively in Section 4.4.
	20d	Present results of all sensitivity analyses conducted to assess the robustness of the synthesized results.	Not applicable — no sensitivity analyses (no quantitative synthesis). Robustness assessed via per-predictor risk of bias and GRADE certainty.
Reporting biases	21	Present assessments of risk of bias due to missing results (arising from reporting biases) for each synthesis assessed.	Section 3.12 (Certainty of Evidence (GRADE)); Section 4.4. GRADE downgrading for inconsistency or indirectness (e.g., PSMA-PET SUVmax, cribriform/IDC) reflects per-predictor judgements; potential for reporting bias acknowledged narratively given absence of formal statistical tests.
Certainty of evidence	22	Present assessments of certainty (or confidence) in the body of evidence for each outcome assessed.	Section 3.12 (Certainty of Evidence (GRADE)). Per-predictor GRADE ratings: moderate (PI-RADS, biopsy approach combined-vs-systematic, PSMA-PET SUVmax, PSAD); low (cribriform/IDC, Decipher, phi, ML models); very low (germline mutations as direct predictors). No predictor reached high certainty.
**DISCUSSION**			
Discussion	23a	Provide a general interpretation of the results in the context of other evidence.	Section 4.1 (Principal Findings); Section 4.2 (Comparison with Prior Literature). Three principal findings articulated and contextualised against Cohen 2008, the Hopkins 2012 series, the Diamand 2025 GG2 meta-analysis, and contemporary mpMRI/PSMA/AI evidence.
	23b	Discuss any limitations of the evidence included in the review.	Section 3.11; Section 3.12; Section 4.4 (limitations on heterogeneity in upgrading definitions across primary studies and on PROBAST analysis-domain concerns flagged in 5 of 8 prediction-model studies, with only 1 of 8 externally validated).
	23c	Discuss any limitations of the review processes used.	Section 4.4 (Strengths and Limitations), limitations 1–3: single-database PubMed search; heterogeneity in upgrading definitions across primary studies precluding quantitative meta-analysis; PROBAST analysis-domain concerns in 5 of 8 prediction-model studies.

PRISMA

PRISMA 2020 Checklist

Section and Topic	Item #	Checklist item	Location where item is reported
	23d	Discuss implications of the results for practice, policy, and future research.	Section 4.3 (Clinical Implications) for practice: active surveillance, treatment selection, and biopsy strategy. Section 4.5 (Future Directions) for research: multimodal modelling; prospective external validation; geographic representation; multi-database successor synthesis.
**OTHER INFORMATION**			
Registration and protocol	24a	Provide registration information for the review, including register name and registration number, or state that the review was not registered.	Section 2.1 (Protocol and Registration). The review was prospectively registered with PROSPERO on 25 April 2026 (registration number CRD420261378765; https://www.crd.york.ac.uk/PROSPERO/view/CRD420261378765).
	24b	Indicate where the review protocol can be accessed, or state that a protocol was not prepared.	Section 2.1. Protocol developed a priori following PRISMA-P recommendations and registered with PROSPERO; the protocol summary is available via the PROSPERO registration record. The full protocol is available from the corresponding author on reasonable request.
	24c	Describe and explain any amendments to information provided at registration or in the protocol.	Section 2.1. No protocol amendments are reported in the manuscript; if amendments occurred during conduct, they will be transparently documented at submission with rationale and date.
Support	25	Describe sources of financial or non-financial support for the review, and the role of the funders or sponsors in the review.	Funding statement (back matter): "This research received no external funding." No funder or sponsor had any role in the conduct of the review. Publication costs will be supported by Victor Babes University of Medicine and Pharmacy Timisoara, which had no role in study selection, data extraction, risk-of-bias assessment, synthesis, or interpretation.
Competing interests	26	Declare any competing interests of review authors.	Conflicts of Interest statement (back matter): the authors declare no commercial conflicts of interest. Authors affiliated with Victor Babes University of Medicine and Pharmacy Timisoara disclose that one of the included primary studies (Pasecinic et al. 2025) was produced by this institution; this self-citation was included on scientific merits, was identified as eligible during full-text screening against the pre-specified criteria, and was independently risk-of-bias assessed using the QUIPS tool by reviewers not affiliated with the source-study author group (see Supplementary File S2).
Availability of data, code and other materials	27	Report which of the following are publicly available and where they can be found: template data collection forms; data extracted from included studies; data used for all analyses; analytic code; any other materials used in the review.	Data Availability Statement (back matter); Section 2.7 (Software). All data supporting the findings are available within the article, in the cited primary studies, and in the supplementary materials (Supplementary File S1, PubMed search strategy; Supplementary File S2, per-study QUIPS risk-of-bias domain ratings; Supplementary File S3, per-study PROBAST risk-of-bias and applicability domain ratings; Supplementary File S4, per-study QUADAS-2 risk-of-bias and applicability domain ratings). The data-extraction file and analytic code for figure generation (Python 3.11 with matplotlib) are available from the corresponding author on reasonable request.

From: Page MJ, McKenzie JE, Bossuyt PM, Boutron I, Hoffmann TC, Mulrow CD, et al. The PRISMA 2020 statement: an updated guideline for reporting systematic reviews. BMJ 2021;372:n71. doi: 10.1136/bmj.n71. This work is licensed under CC BY 4.0. To view a copy of this license, visit https://creativecommons.org/licenses/by/4.0/
