# Supplementary material for: ISUP Grade Group Migration Between Prostate Biopsy and Radical Prostatectomy: A Systematic Review of Emerging Predictors
Source: Biomedicines. 2026 Jul 31;14(8):1736. doi: 10.3390/biomedicines14081736 (PMC13509513; doi:10.3390/biomedicines14081736)
Supplement: Supplementary file 1 [file biomedicines-14-01736-s001.zip › Supplementary_File_S1_Search_Strategy - revised.pdf]

Supplementary File S1
PubMed/MEDLINE Search Strategy

ISUP Grade Group Migration Between Prostate Biopsy and Radical Prostatectomy: A Systematic Review on Emerging Predictors

1. Database, dates, and platform

Database: MEDLINE, accessed via the PubMed interface (https://pubmed.ncbi.nlm.nih.gov).

Search window: 1 January 2010 to 5 May 2026 (final search update).

Initial search executed: 25 April 2026.

Final update search: 5 May 2026.

Language restriction: English-language publications only.

Other databases searched: None. Embase, the Cochrane Central Register of Controlled Trials (CENTRAL), Scopus, and Web of Science were not searched. No preprint servers, trial registries, or conference proceedings were systematically interrogated. Single-database searching is acknowledged as a limitation in Section 4.4 of the main manuscript.

Supplementary searching: Citation searching of eight contemporary systematic reviews and meta-analyses [refs 13, 16, 22, 27, 28, 37, 44, 45 of the main manuscript] yielded an additional 38 records that were subjected to the same eligibility criteria as records identified through the database search.

2. Conceptual block structure

The search strategy was constructed using three conceptual blocks linked by AND, with synonyms within each block linked by OR. Medical Subject Headings (MeSH) terms were combined with free-text terms in title and abstract fields. Truncation was applied where biologically appropriate using the PubMed wildcard operator (*).

The three conceptual blocks were:

- **Block 1 — Population:** prostate adenocarcinoma and radical prostatectomy.
- **Block 2 — Grading system and migration event:** Gleason score, Grade Group, ISUP, upgrading, downgrading, discordance, concordance, reclassification, upstaging.
- **Block 3 — Diagnostic or prognostic intervention:** biopsy approach, multiparametric MRI, PI-RADS, PSMA-PET, tissue and serum and urinary biomarkers of interest, genomic classifiers, deep learning, radiomics.

3. Full PubMed search string (verbatim)

The verbatim PubMed search string executed on 25 April 2026 and updated on 5 May 2026 is reproduced below. Line numbering is added for reference and was not part of the executed query. The query was entered as a single string with the three blocks linked by AND.

3.1. Block 1 — Population

```
#1   "Prostatic Neoplasms"[MeSH Terms]
#2   "Adenocarcinoma"[MeSH Terms]
#3   prostate cancer[Title/Abstract]
#4   prostate carcinoma[Title/Abstract]
#5   prostate adenocarcinoma[Title/Abstract]
#6   prostatic neoplasm*[Title/Abstract]
#7   #1 OR #2 OR #3 OR #4 OR #5 OR #6

#8   "Prostatectomy"[MeSH Terms]
#9   radical prostatectomy[Title/Abstract]
#10  prostatectomy[Title/Abstract]
#11  RARP[Title/Abstract]
#12  RALP[Title/Abstract]
#13  robot-assisted prostatectomy[Title/Abstract]
#14  #8 OR #9 OR #10 OR #11 OR #12 OR #13

#15  #7 AND #14   [Block 1 result]
```

3.2. Block 2 — Grading system and migration event

```
#16  "Neoplasm Grading"[MeSH Terms]
#17  Gleason[Title/Abstract]
#18  Gleason score[Title/Abstract]
#19  Gleason grade[Title/Abstract]
#20  Gleason pattern[Title/Abstract]
#21  Grade Group[Title/Abstract]
#22  ISUP[Title/Abstract]
#23  International Society of Urological Pathology[Title/Abstract]
#24  cribriform[Title/Abstract]
#25  intraductal carcinoma[Title/Abstract]
#26  IDC-P[Title/Abstract]

#27  upgrad*[Title/Abstract]
#28  downgrad*[Title/Abstract]
#29  upstag*[Title/Abstract]
```

```
#30  downstag*[Title/Abstract]
#31  reclassif*[Title/Abstract]
#32  concordan*[Title/Abstract]
#33  discordan*[Title/Abstract]
#34  agreement[Title/Abstract]
#35  pathological correlation[Title/Abstract]
#36  biopsy-prostatectomy[Title/Abstract]

#37  (#16 OR #17 OR #18 OR #19 OR #20 OR #21 OR #22 OR #23 OR #24 OR #25 OR #26)
#38  (#27 OR #28 OR #29 OR #30 OR #31 OR #32 OR #33 OR #34 OR #35 OR #36)
#39  #37 AND #38   [Block 2 result]
```

3.3. Block 3 — Diagnostic or prognostic intervention

```
#40  "Image-Guided Biopsy"[MeSH Terms]
#41  "Biopsy, Large-Core Needle"[MeSH Terms]
#42  prostate biopsy[Title/Abstract]
#43  systematic biopsy[Title/Abstract]
#44  targeted biopsy[Title/Abstract]
#45  MRI-targeted biopsy[Title/Abstract]
#46  fusion biopsy[Title/Abstract]
#47  in-bore biopsy[Title/Abstract]
#48  transrectal[Title/Abstract]
#49  transperineal[Title/Abstract]
#50  TRUS[Title/Abstract]
#51  cognitive fusion[Title/Abstract]

#52  "Magnetic Resonance Imaging"[MeSH Terms]
#53  multiparametric MRI[Title/Abstract]
#54  mpMRI[Title/Abstract]
#55  PI-RADS[Title/Abstract]
#56  Prostate Imaging Reporting and Data System[Title/Abstract]
#57  diffusion-weighted[Title/Abstract]
#58  apparent diffusion coefficient[Title/Abstract]
#59  ADC[Title/Abstract]
#60  T2-weighted[Title/Abstract]

#61  "Positron Emission Tomography Computed Tomography"[MeSH Terms]
#62  PSMA[Title/Abstract]
#63  prostate-specific membrane antigen[Title/Abstract]
```

#64 PSMA-PET[Title/Abstract]

#65 68Ga-PSMA[Title/Abstract]

#66 18F-PSMA[Title/Abstract]

#67 SUVmax[Title/Abstract]

#68 PRIMARY score[Title/Abstract]

#69 Decipher[Title/Abstract]

#70 Prolaris[Title/Abstract]

#71 Oncotype[Title/Abstract]

#72 genomic classifier[Title/Abstract]

#73 Genomic Prostate Score[Title/Abstract]

#74 ExoDx[Title/Abstract]

#75 MyProstateScore[Title/Abstract]

#76 SelectMDx[Title/Abstract]

#77 PCA3[Title/Abstract]

#78 4Kscore[Title/Abstract]

#79 Prostate Health Index[Title/Abstract]

#80 PHI density[Title/Abstract]

#81 PHID[Title/Abstract]

#82 BRCA1[Title/Abstract] OR BRCA2[Title/Abstract]

#83 ATM[Title/Abstract]

#84 HOXB13[Title/Abstract]

#85 CHEK2[Title/Abstract]

#86 MSH2[Title/Abstract]

#87 PTEN[Title/Abstract]

#88 germline[Title/Abstract]

#89 DNA damage repair[Title/Abstract]

#90 DDR gene*[Title/Abstract]

#91 polygenic risk score[Title/Abstract]

#92 "Artificial Intelligence"[MeSH Terms]

#93 "Deep Learning"[MeSH Terms]

#94 artificial intelligence[Title/Abstract]

#95 deep learning[Title/Abstract]

#96 machine learning[Title/Abstract]

#97 convolutional neural network*[Title/Abstract]

#98 radiomics[Title/Abstract]

#99 digital pathology[Title/Abstract]

```
#100 whole-slide imag*[Title/Abstract]
#101 Paige Prostate[Title/Abstract]
#102 PANDA challenge[Title/Abstract]

#103 (#40 OR #41 OR #42 OR #43 OR #44 OR #45 OR #46 OR #47 OR #48 OR #49 OR #50 OR #51)
#104 (#52 OR #53 OR #54 OR #55 OR #56 OR #57 OR #58 OR #59 OR #60)
#105 (#61 OR #62 OR #63 OR #64 OR #65 OR #66 OR #67 OR #68)
#106 (#69 OR #70 OR #71 OR #72 OR #73 OR #74 OR #75 OR #76 OR #77 OR #78 OR #79 OR #80 OR #81)
#107 (#82 OR #83 OR #84 OR #85 OR #86 OR #87 OR #88 OR #89 OR #90 OR #91)
#108 (#92 OR #93 OR #94 OR #95 OR #96 OR #97 OR #98 OR #99 OR #100 OR #101 OR #102)
#109 #103 OR #104 OR #105 OR #106 OR #107 OR #108   [Block 3 result]
```

3.4. Final combined query

```
#110 #15 AND #39 AND #109

#111 #110 AND ("2010/01/01"[Date - Publication] : "2026/05/05"[Date - Publication])

#112 #111 AND English[Language]
```

The result of #112 was the final record set exported from PubMed for screening (n = 847).

4. Filters and operational decisions

- **Date filter:** applied as a publication-date range using the [Date - Publication] field tag (line #111). The range was 1 January 2010 to 5 May 2026.
- **Language filter:** applied using the English[Language] tag (line #112).
- **Methodological filters:** no methodological filter (e.g., Cochrane Highly Sensitive Search Strategy, Haynes clinical-query filters) was applied, in order to retain observational study designs (cohort studies, case-control studies, registry analyses) relevant to a prognostic-factor and prediction-model review.
- **Article-type filter:** no article-type filter was applied at search time. Editorials, narrative reviews, letters, conference abstracts without accessible full text, and case reports were excluded at the screening stage rather than at the search stage to preserve sensitivity.
- **Truncation:** the PubMed wildcard operator (*) was used for biologically appropriate stems (e.g., upgrad*, downgrad*, reclassif*, concordan*, discordan*, neoplasm*, gene*, imag*). PubMed truncation requires at least four characters before the wildcard and was applied accordingly.

- **Field tags:** Title/Abstract searching ([Title/Abstract]) was used for free-text terms; MeSH ([MeSH Terms]) was used where a controlled-vocabulary term was available. Subheadings and explosion were used at PubMed defaults.
- **Operators:** Boolean AND, OR, and NOT were used. NOT was not applied to exclude topics, in order to avoid inadvertent loss of relevant records. Adjacency operators were not used as PubMed does not support them in the same form as Embase.

5. Hit counts by block and per the final query

Hit counts are reported as recorded at the time of the final search update on 5 May 2026. Counts may differ slightly from those obtainable on later re-execution because the MEDLINE index is updated continuously.

Line	Block / sub-block	Records
#15	Block 1 — Population (prostate cancer AND prostatectomy)	48,213
#39	Block 2 — Grading system AND migration event	11,847
#103	Block 3a — Biopsy approach	28,419
#104	Block 3b — Multiparametric MRI / PI-RADS	9,372
#105	Block 3c — PSMA-PET	3,158
#106	Block 3d — Tissue / serum / urinary biomarkers	4,021
#107	Block 3e — Germline alterations	2,694
#108	Block 3f — Artificial intelligence / radiomics / digital pathology	3,816
#109	Block 3 (combined sub-blocks 3a–3f)	44,712
#110	Blocks 1 AND 2 AND 3 (no date or language filter)	1,164
#111	Date-limited (1 January 2010 – 5 May 2026)	962
#112	Final exported set (English-language, 2010–2026)	847

Note. *The 847-record figure in the final row matches the value reported in Section 3.1 and Figure 1 of the main manuscript. The 38 additional records identified through citation searching of eight prior systematic reviews [refs 13, 16, 22, 27, 28, 37, 44, 45 of the main manuscript] were added to this set before deduplication, yielding the 885 records that entered the deduplication step (885 – 23 duplicates = 862 records screened).*

6. Citation searching of prior systematic reviews

Forward and backward citation searching was performed on the reference lists and on Web-of-Science forward-citation reports of the eight prior systematic reviews and meta-analyses listed below. Records identified through citation searching were screened against the same eligibility criteria as records identified through the PubMed search.

Ref.	Prior systematic review or meta-analysis	Predictor domain
[13]	Cohen MS et al. (2008). Comparing the Gleason prostate biopsy and Gleason prostatectomy grading system. Eur Urol 54:371–381.	Concordance / migration overall
[16]	Wu S. et al. (2026). Gleason Score 3 + 4 (Grade Group 2) Prostate Cancer on Biopsy and Postoperative Pathological Upgrading: A Systematic Re-view and Meta-Analysis. Clin Genitourin Cancer 2026, 24 (1), 102461.	GG2 upgrading
[22]	Weinstein IC et al. (2023). Impact of MRI targeting on pathologic upgrading and downgrading at prostatectomy. Eur Urol Oncol 6:355–365.	Biopsy approach
[27]	Goel S et al. (2020). Concordance Between Biopsy and Radical Prostatectomy Pathology in the Era of Targeted Biopsy. Eur Urol Oncol 3:10–20.	Biopsy approach
[28]	Chen DC et al. (2025). Impact of intraprostatic PSMA maximum standardized uptake value following prostatectomy. BJU Int 135:720–732.	PSMA-PET SUVmax
[37]	Wang Y et al. (2023). Predictive Factors for Gleason Score Upgrading After Radical Prostatectomy. Urol Int 107:460–479.	Clinical predictors
[47]	Wang X et al. (2021). Old men with prostate cancer have higher risk of Gleason score upgrading and pathological upstaging after initial diagnosis. World J Surg Oncol 19:18.	Age and clinical predictors
[48]	Liu Y et al. (2025). The predictive value of PSMA PET/CT in determining pathological upgrading of prostate cancer: A pooling up analysis. Front Oncol 15:1525890.	PSMA-PET pooled estimates

Citation searching yielded 38 unique additional records that were not retrieved by the PubMed query but were judged at least potentially eligible at title screening. These were added to the records identified through PubMed/MEDLINE searching before deduplication.

7. Deviations from the registered protocol and limitations

Protocol-search alignment: the executed search strategy matches the strategy described in the prospectively registered PROSPERO protocol (registration number CRD420261378765).

Single-database limitation: we acknowledge that single-database searching of PubMed/MEDLINE falls below the multi-database threshold recommended by PRISMA 2020 item 7 and the Cochrane Handbook §4.3.2 for clinical questions of this scope. Embase in particular

indexes a substantial fraction of European urology and pathology output that is not consistently captured in MEDLINE; some primary studies and prediction-model reports relevant to biopsy-to-radical-prostatectomy grade migration are likely to have been missed. This limitation is discussed in Section 4.4 of the main manuscript and motivates a multi-database successor synthesis (MEDLINE, Embase, CENTRAL, Scopus, Web of Science).

Grey literature: trial registries (ClinicalTrials.gov, WHO ICTRP), conference proceedings (American Urological Association, European Association of Urology, American Society of Clinical Oncology, Genitourinary Cancers Symposium), and preprint servers (medRxiv, bioRxiv) were not systematically searched.

Author contact: corresponding authors of included studies were not contacted to request unpublished data, additional outcomes, or clarification of reported results. Only data reported in the published full text or in the supplementary materials of included studies were extracted.

Reproducibility: the search strategy reported here is sufficient to reproduce the search in PubMed. Counts on re-execution will differ slightly from those reported in Section 5 because the MEDLINE index is updated continuously and because PubMed automated term mapping may evolve over time.

8. How to reproduce the search

To reproduce the executed search:

- Navigate to the PubMed Advanced Search interface (https://pubmed.ncbi.nlm.nih.gov/advanced/).
- Build each block (Section 3.1, 3.2, 3.3) as a separate Search Builder query, using the field tags shown in this document.
- Combine the three block results in History and Search Details using the AND operator (line #110).
- Apply the publication-date filter for 1 January 2010 to 5 May 2026 (line #111).
- Apply the English-language filter (line #112).
- Export the final result set as a .ris or .nbib file for import into the reference-management software of choice.

The exported records can then be deduplicated and screened in Rayyan or any equivalent screening platform.
